# Coordinated cellular architecture reveals weakened cortical structure–behavior coupling in normal aging

**DOI:** 10.3389/fnagi.2026.1932616

**Published:** 2026-09-30

**Authors:** Xiaoyi Zhan, Pengcheng Tan, Tirzah Tan, Chia-Chien Chen

**Affiliations:** 1 Behavioral Science Program, Division of Natural and Applied Sciences, Duke Kunshan University, Suzhou, Jiangsu, China; 2 Molecular Bioscience Program, Division of Natural and Applied Sciences, Duke Kunshan University, Suzhou, Jiangsu, China; 3 Kunshan First People’s Hospital, Kunshan, Jiangsu, China; 4 Department of Molecular Cell and Developmental Biology, University of California, Santa Cruz, Santa Cruz, CA, United States

**Keywords:** barrel cortex, cortical aging, dendritic architecture, microglial morphology, sensory-guided behavior, structure–behavior coupling

## Abstract

Normal aging compromises behavioral functions supported by cortical circuits, yet how age-related cellular reorganization relates to weakened circuit-behavior correspondence remains poorly defined. Here, we examined layer 4 (L4) of the mouse primary somatosensory barrel cortex, a thalamorecipient lamina in which dendritic organization, microglial process architecture, and whisker-dependent tactile function can be analyzed within the same sensory system. Aged mice showed impaired whisker-dependent texture discrimination. Using high-resolution three-dimensional cellular reconstructions, we quantified the morphology and spatial architecture of L4 pyramidal neurons and microglia in young and aged mice. We then applied within-domain principal component analysis (PCA) to derive architecture scores and tested how these scores mapped onto whisker-dependent behavior. Aging reduced the complexity and spatial extent of L4 pyramidal dendritic arbors, while microglia showed reduced process complexity and territorial occupancy. Across both cell types, these changes converged on a more spatially restricted local cellular architecture. PCA organized high-dimensional morphometric variables into dominant architecture scores that captured coordinated morphometric organization within the neuronal and microglial domains. In young animals, dominant architecture scores were associated with whisker-dependent tactile discrimination; residual scores and same-texture encoding indices did not show significant corresponding association. Within-age permutation analyses and direct Fisher r-to-z comparisons supported an age-dependent weakening of architecture–behavior correspondence. These findings identify parallel neuronal and microglial morphological changes as an anatomical axis of cortical aging, with restricted cellular architecture accompanied by weakened architecture-behavior correspondence in sensory cortex.

## 1 Introduction

Normal aging is accompanied by gradual changes in sensory processing, attention, learning, memory, and adaptive behavioral flexibility across humans and experimental animals (Mather, 2010; Alexander et al., 2012; Bizon et al., 2012; Lee and Kim, 2022; Dexter and Ossmy, 2023), yet the cellular architectures that underlie these age-related functional changes remain incompletely understood. Recent work increasingly emphasizes that brain aging involves coordinated reorganization across cell types and brain regions (Soreq et al., 2017; Ximerakis et al., 2019). Although synaptic inputs are central to neural computation, the capacity of a neuron to receive, distribute, and integrate those inputs depends first on the existence, geometry, and spatial organization of its dendritic arbor. Dendritic arbors define the physical territory within which synaptic inputs can be sampled, compartmentalized, and integrated into neuronal output (Häusser et al., 2000; London and Häusser, 2005; Spruston, 2008). Dendritic architecture therefore represents a necessary structural condition for circuit-level processing because it determines the local spatial domain over which synaptic inputs can contribute to neuronal processing.

This structural logic extends beyond neurons, as the same cortical territory is also occupied, surveyed, and regulated by glial cells whose morphology determines how they interact with the local tissue environment. Microglia constitute an additional glial domain within this local cellular environment, as their processes continuously survey the brain parenchyma, form direct interactions with synaptic and somatic neuronal compartments, and respond to changes in neuronal activity and tissue state (Nimmerjahn et al., 2005; Tremblay et al., 2010; Schafer et al., 2012; Badimon et al., 2020; Cserép et al., 2020). Importantly, recent lifespan and aging studies further show that microglia undergo regionally and temporally organized changes in morphology, transcriptomic state, and surveillance properties during normal aging (Menassa et al., 2022; Godeanu et al., 2023; Li et al., 2023; Tieu et al., 2025). Quantitative cellular morphometrics therefore provides a fundamental and unbiased way to describe the actual architecture of the cortical cellular environment, including the dendritic space available for synaptic input and the glial process field available for tissue surveillance and interaction, and to test whether aging weakens the correspondence between cellular architecture and sensory-guided behavior.

The rodent whisker-to-barrel system provides an ideal model for examining how cellular architecture relates to sensory-guided behavior because it contains a well-defined peripheral sensory apparatus, a highly organized ascending pathway, a discrete cortical representation, and measurable behavioral outputs (Woolsey and Van Der Loos, 1970; Petersen, 2007; Feldmeyer et al., 2013). Within this system, layer 4 (L4) of the barrel cortex is especially important because it contains the most recognizable barrel structures and serves as the principal thalamorecipient lamina for whisker-derived inputs, where afferent sensory information first enters the cortical circuit for subsequent processing (Petersen, 2007; Wimmer et al., 2010; Feldmeyer et al., 2013). L4 excitatory neurons, in turn, provide a local cellular substrate through which incoming sensory information can be organized and distributed within cortical circuits. Prior work has shown that sensory experience during early development affects both structure and function in this system. Whisker deprivation changes cortical physiology and receptive field properties, alters whisker-dependent texture discrimination, and modifies multiple cellular and extracellular features of the barrel cortex, including dendritic architecture, dendritic protrusions, perineuronal nets, myelin, and microglia (Simons and Land, 1987; Carvell and Simons, 1996; Zuo et al., 2005; McRae et al., 2007; Yang et al., 2009; Chen et al., 2012, 2015; Barrera et al., 2013; Kalambogias et al., 2020; Chen and Brumberg, 2021). Similar sensory experience–dependent relationships have also been described in other sensory cortical areas, including the primary visual and auditory cortices (Wiesel and Hubel, 1963; Hubel and Wiesel, 1970; Recanzone et al., 1993; Zhang et al., 2001). Together, these findings support a structural-functional-behavioral framework in which sensory experience shapes cortical cellular architecture, this architecture in turn supports sensory processing, and disruption of this organization alters sensory-guided behavior. More recent microglial morphometric work in somatosensory cortex and across species further supports the interpretation that microglial architecture reflects the capacity of these cells to occupy, survey, and interact with local tissue environments (Carvalho-Paulo et al., 2021; Godeanu et al., 2023; Reddaway et al., 2023; Green and Rowe, 2024).

Despite extensive work on experience-dependent plasticity in the barrel cortex (Zuo et al., 2005; McRae et al., 2007; Yang et al., 2009; Chen et al., 2012, 2015; Kalambogias et al., 2020; Chen and Brumberg, 2021) and prior aging studies of cortical neuronal morphology (Coleman et al., 1986; Dickstein et al., 2007, 2013), it remains less clear how normal aging affects the neuronal and glial architecture within L4, and whether these cellular features are associated with variation in behavior. Here, we examined age-related changes in L4 barrel cortex by combining neuronal and microglial morphometry with whisker-dependent behavioral analysis, correlation mapping, and principal component analysis (PCA). Rather than treating individual morphometric measurements as isolated endpoints, we organized them into broader architectural domains that define the structure of the local cortical environment. These domains reflect the dendritic organization available for synaptic integration, the glial process architecture available for tissue surveillance and interaction, and the spatial occupancy through which these cellular elements may support circuit-level function. Such an approach is important because age-related behavioral decline may reflect concomitant shifts in the local structural organization within which neurons and glia jointly support cortical function. Recent work has similarly emphasized the need to move beyond single-parameter descriptions of microglial form toward higher-dimensional and reproducible morphometric analyses that better capture complex cellular states (Reddaway et al., 2023; Green and Rowe, 2024; Kim et al., 2024). Using high-dimensional morphometric datasets, we asked whether neuronal and glial features reveal parallel aging-related structural patterns associated with behavioral performance. This framework allowed us to examine whether normal aging reorganizes local cortical architecture and alters its correspondence with sensory-guided behavior.

## 2 Methods

### 2.1 Animals

C57BL/6J mice were used in the present study. Young (50 days to 2 months old) and a subset of aged mice (18–20 months old) were born in a local Charles River-derived colony; additional aged mice were obtained from NIA aged colony and acclimated for ≥3 weeks before behavioral testing and subsequent tissue collection. Mice of both sexes were housed in standard cages under a 12 h light/dark cycle with *ad libitum* access to food and water. Mice were grouped by age cohort at the time of behavioral testing and subsequent sacrifice for brain retrieval. All animal procedures complied with institutional guidelines for the care and use of laboratory animals and were approved by the Institutional Animal Care and Use Committees of Duke Kunshan University and the University of California, Santa Cruz.

### 2.2 Golgi staining

Golgi impregnation of cortical neurons was performed using the FD Rapid GolgiStain kit (FD NeuroTechnologies, Columbia, MD), following established protocols for cortical neuronal labeling (Chen et al., 2009, 2012, 2015; Zemmar et al., 2018). Briefly, mice were deeply anesthetized with Euthasol, and brains were rapidly removed without transcardial perfusion, consistent with the manufacturer’s protocol. Brains were immersed in impregnation solution consisting of equal volumes of Solutions A and B for 12 days at room temperature. Brains were then transferred into cryoprotectant solution (Solution C) and stored at 4 °C for at least 7 days. Coronal sections containing S1BF were cut at 200 μm using a cryostat, mounted onto gelatin-coated slides, and processed in developing solution prepared according to the manufacturer’s instructions. Sections were then dehydrated through graded ethanol solutions, cleared in SafeClear xylene substitute (StatLab, McKinney, TX), and coverslipped with Permount. Golgi impregnation was used because it permits sparse labeling of neuronal somata and dendritic arbors suitable for detailed three-dimensional reconstruction of cortical pyramidal neurons (Chen et al., 2009, 2012, 2015; Zemmar et al., 2018).

### 2.3 IBA1 immunohistochemistry

Microglia were labeled using IBA1 immunohistochemistry with an avidin-biotin horseradish peroxidase method and 3,3′-diaminobenzidine (DAB)-nickel chromogen, as previously described for barrel cortex microglial morphology (Kalambogias et al., 2020). Briefly, deeply anesthetized mice were transcardially perfused with 0.01 M phosphate-buffered saline (PBS) followed by 4% paraformaldehyde, and brains were removed, post-fixed overnight, cryoprotected in 30% sucrose in 0.01 M PBS, and sectioned coronally at 50 μm using a Leica vibratome. Free-floating sections were washed in 0.01 M PBS, quenched for endogenous peroxidase activity in 1% H2O2 and 0.5% methanol, permeabilized with 0.7% Triton X-100, and blocked with 10% normal donkey serum (Jackson ImmunoResearch, 017-000-121). Sections were then incubated with goat anti-IBA1 primary antibody (1:1000; Abcam, ab5076), followed by biotinylated donkey anti-goat secondary antibody (1:500; Jackson ImmunoResearch, 705-065-147) and avidin-biotin horseradish peroxidase complex (Vector Laboratories, PK-6100). Immunoreactivity was visualized using the DAB Substrate Kit, Peroxidase (HRP), with Nickel (Vector Laboratories, SK-4100), according to the manufacturer’s instructions. Sections were mounted onto positively charged slides, dehydrated through graded ethanol, cleared in SafeClear xylene substitute (StatLab, McKinney, TX), and coverslipped with Permount. Negative control sections processed without primary antibody were included to verify labeling specificity.

### 2.4 Neurolucida reconstruction and morphometric analysis of neuronal and microglial cellular architecture

Golgi-labeled L4 pyramidal neurons and IBA1-labeled microglia were examined and manually reconstructed using an Olympus BX63 microscope system equipped with Neurolucida software. Cells were initially examined at lower magnifications (4× air, NA 0.16) to identify S1BF, L4, and candidate cells for reconstruction. Detailed reconstructions were performed using a high-magnification oil-immersion objective (60× oil, NA 1.42). Reconstructions were performed using Neurolucida 360 or Neurolucida 11.0 software, and morphometric variables were quantified using NeuroExplorer (MBF Biosciences, Williston, VT). This reconstruction-based approach follows prior cortical morphometry studies in which the Neurolucida system was used to quantify somatic morphology, dendritic or process length, branching complexity, Sholl intersections, and related morphometric parameters (Chen et al., 2009, 2012, 2015; Kalambogias et al., 2020; Zemmar et al., 2018).

The primary somatosensory barrel field (S1BF) was identified according to cytoarchitectonic criteria and the characteristic organization of the barrel field, as previously described for Golgi-based studies of the mouse barrel cortex (Chen et al., 2009, 2012, 2015; Valverde, 1998). For Figure 1D, L4 was delineated independently of reconstructed neuronal somata from local barrel-cortex cytoarchitecture; its intersections with the pial-to-white-matter depth axis defined the estimated L4 range, and the same framework was used for microglial localization. L4 pyramidal neurons were selected based on somatic location within L4, complete Golgi impregnation, clear dendritic labeling, pyramidal somatic morphology, tapered and non-truncated dendritic arbors, and the presence of an apical dendrite that typically extended toward layers 2/3, consistent with prior criteria used to identify L4 pyramidal neurons in barrel cortex (Chen et al., 2015). Spiny stellate cells and other L4 excitatory neurons without a clearly identifiable apical dendrite were not included in the neuronal reconstruction dataset. IBA1-labeled microglia were reconstructed from anatomically matched L4 regions of S1BF when the soma and associated processes were clearly labeled and sufficiently isolated for complete reconstruction. Neurons or microglia were excluded if they showed incomplete labeling, substantial overlap with neighboring labeled cells, or truncated dendritic or microglial processes.

**Figure 1:**
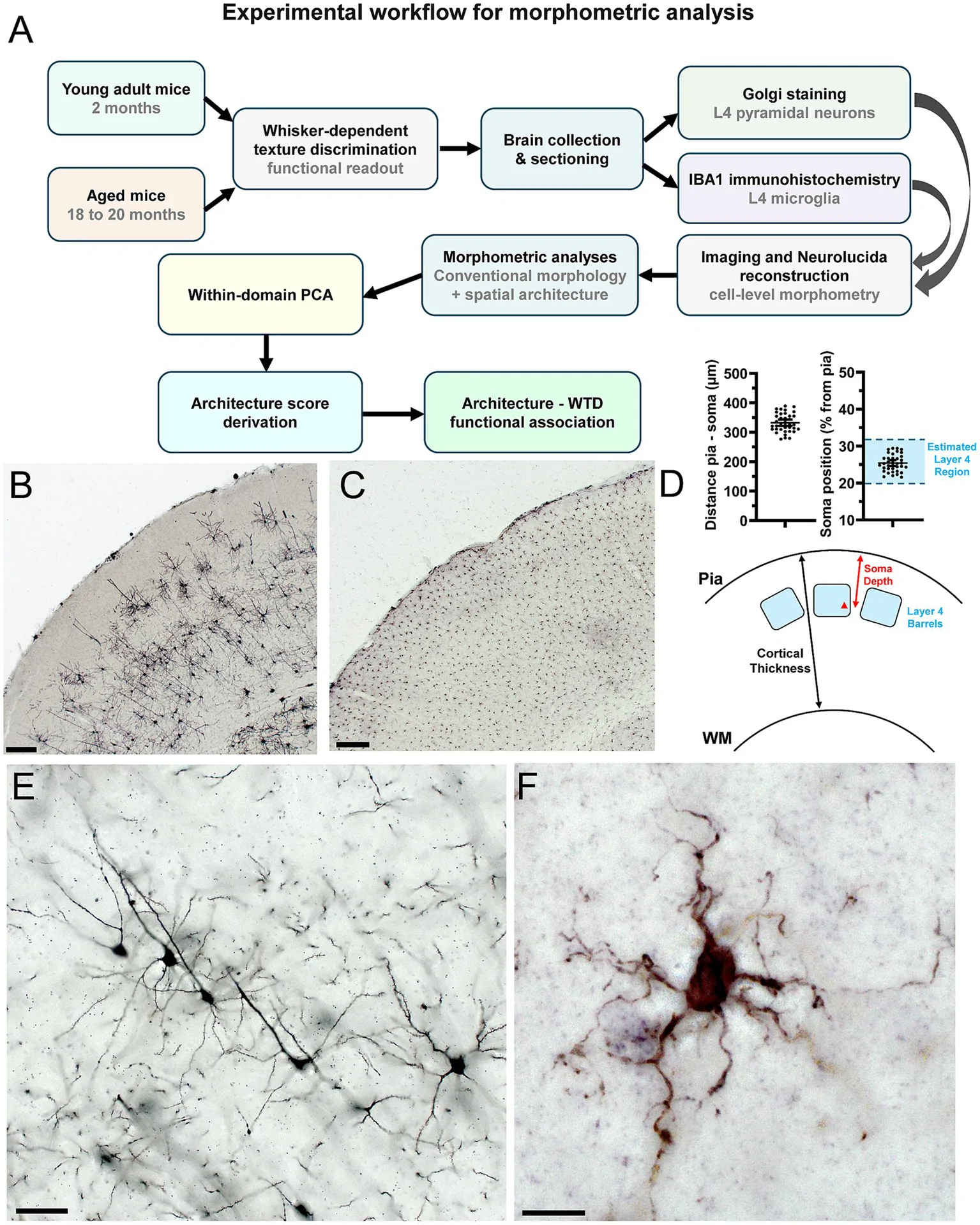
Experimental workflow and anatomical validation of L4 sampling for morphometric analysis. **(A)** Workflow showing the progression from age-group comparison and whisker-dependent texture discrimination testing to Golgi- and IBA1-based cellular reconstruction, morphometric analysis, principal component analysis, and architecture score association with behavior. **(B)** Representative low-magnification (4×) image of Golgi-stained S1BF showing the cortical region used for neuronal sampling. **(C)** Representative low-magnification (4×) image of IBA1-stained S1BF showing microglial labeling in an anatomically matched region. **(D)** Quantification of soma position for reconstructed neurons. Soma depth was measured from the pial surface to the center of the soma and expressed as a percentage of total cortical thickness. Reconstructed somata were located within the estimated L4 region of S1BF. Bottom: Schematic illustration of soma-depth measurement relative to the pia, white matter, and L4 barrels. Each dot represents one reconstructed neuronal soma; data are shown as mean ± SD. **(E)** Representative image of Golgi-labeled L4 pyramidal neurons obtained at 20× magnification. **(F)** Representative image of an IBA1-labeled microglial cell obtained at 60× magnification. Scale bars: 100 μm in **(B,C)**, 50 μm in **(E)**, and 10 μm in **(F)**.

Morphometric variables were organized into two major geometric domains: *Conventional Morphology* and *Spatial Architecture*. *Conventional Morphology* included traditional somatic and arbor measurements, including somatic size-related measures, dendritic or microglial process length, branching measures, node and endpoint measures, and surface area- and volume-related measures. For neuronal analyses, dendritic variables were quantified separately for apical dendrites, basal dendrites, and total dendritic arbors, consistent with prior compartment-specific dendritic morphometric analyses (Chen et al., 2009, 2012; Zemmar et al., 2018). These variables were used to quantify overall cellular size and branching complexity, consistent with previously established reconstruction-based morphometric analyses (Chen et al., 2009, 2012, 2015; Kalambogias et al., 2020; Zemmar et al., 2018).

*Spatial Architecture* included measurements describing how cellular arbors were distributed within space. This domain combined Sholl-based radial distribution, convex hull-based territorial occupancy, and polarity-based directional organization. Sholl variables quantified soma-centered radial structure, including intersections and dendritic or process length across radial distance, following the original Sholl framework and its later applications in cortical morphometry (Chen et al., 2009, 2012, 2015; Sholl, 1956; Zemmar et al., 2018). Convex hull variables quantified the global territory occupied by the cellular arbor, including hull area, perimeter, volume, and surface area. Polarity variables quantified directional bias relative to anatomical reference axes using a morphometric approach previously described (Chen et al., 2009). For L4 pyramidal neurons, the direction of the apical dendrite was defined as 0°, and the direction toward the estimated barrel center was defined as 90°. The barrel-center direction was estimated from local barrel cytoarchitecture and the established orientation of L4 spiny stellate dendrites toward the barrel hollow (Harris and Woolsey, 1981; Lübke et al., 2000). These spatial architecture variables were analyzed together because they represent related aspects of soma-centered and anatomically anchored arbor organization that are not captured by conventional size- and branching-based measures.

### 2.5 Within-domain intercorrelation analysis and variable curation

Within each morphometric domain, intercorrelation matrices were generated for neuronal *Conventional Morphology*, neuronal *Spatial Architecture*, microglial *Conventional Morphology*, and microglial *Spatial Architecture*. Pearson or Spearman coefficients were used according to variable distribution and the presence of influential outliers. Correlation matrices were used to identify redundant variables before PCA, following prior morphometric classification work and standard PCA practice aimed at avoiding double-weighting of overlapping structural measurements (Chen et al., 2009; Jolliffe and Cadima, 2016). Variables were considered redundant when |*r*| ≥ 0.90 and represented the same biological or mathematical feature. For redundant variable sets, one representative variable was retained based on interpretability, domain relevance, consistency across neuronal and microglial datasets, and lower redundancy with the remaining variables. Variable removal was based only on redundancy and interpretability, independent of age-group differences or behavioral correlations.

### 2.6 Principal component analysis and PCA-derived architecture scores

Principal component analysis was performed using GraphPad Prism version 11. PCA was used as an unsupervised multivariate data-reduction method to identify major axes of morphometric variation within each cellular architecture domain. PCA was performed separately for neuronal *Conventional Morphology*, neuronal *Spatial Architecture*, microglial *Conventional Morphology*, and microglial *Spatial Architecture*. PCA was not performed separately for Sholl, convex hull, or polarity variables alone because these subfamilies were treated as related components of *Spatial Architecture* rather than isolated domains.

Before PCA, each morphometric variable was standardized using z-score transformation so that variables measured on different scales contributed comparably to the analysis, consistent with standard PCA practice (Jolliffe and Cadima, 2016). Pooled cell-level data were used to construct the morphometric PCA input matrix because the purpose of PCA was to identify the dominant axes of cellular variation across the reconstructed population. Cell-level PCA scores were used to visualize reconstructed-cell distributions. For animal-level summaries and behavior-related analyses, cell-level scores were averaged within each animal before statistical testing or correlation analysis.

Principal components were retained using the Kaiser criterion, with scree plot inspection and cumulative variance explained used to confirm the retained component structure (Cattell, 1966; Kaiser, 1960). Component loadings were used for descriptive interpretation, with absolute loading values ≥0.40 treated as meaningful, ≥0.50 as strong, and ≥0.70 as dominant contributors. Because PCA axis signs are mathematically indeterminate (Bro et al., 2008; Jolliffe and Cadima, 2016), PC orientation was standardized after PCA fitting and component retention. For each PC, if the young-group mean score was lower than the aged-group mean, scores and corresponding loadings were multiplied by −1; otherwise, orientation was unchanged. This aligned PCs to a common young-associated direction before variance-weighted DS/RS construction. Age labels were used only for this post-PCA orientation and not for PCA fitting or retention; WTD was not used in PCA fitting, retention, orientation, weighting, or DS/RS construction.

For each architecture domain, a Dominant Score (DS) was calculated as the variance-weighted sum of retained, oriented PC scores:

w_k_ = variance explained by PC_k_/total variance explained by all PCs included in the combined score

$$\text{Dominant Score}=\Sigma (\text{oriented}\;{\mathrm{PC}}_{k}\times w_{k})$$

For DS, w_k_ was calculated as the variance explained by PC_k_ divided by the total variance explained by all retained PCs included in the DS. A Residual Score (RS) was calculated using the same weighting procedure applied to non-retained PCs:

$$\text{Residual Score}=\Sigma (\text{oriented}\;{\mathrm{PC}}_{k}\times w_{k})$$

For RS, *w*_k_ was calculated relative to the total variance explained by all non-retained PCs included in the residual score. RS was examined as a complementary residual composite to determine whether it showed a corresponding association with WTD performance. Cell-level DS and RS values were averaged within animals for animal-level behavior-related analyses.

### 2.7 Whisker-dependent texture discrimination test

Whisker-dependent texture discrimination (WTD) was adapted from previously described protocols (Chen et al., 2018; Mehdipour et al., 2019, 2021; Wu et al., 2013). Mice were first habituated in an empty arena for 10 min per day for two consecutive days before testing. On the testing day, young mice explored two columns with the same texture during a 5-min encoding phase, whereas aged mice were given a 10-min encoding phase to accommodate slower exploratory behavior and ensure sufficient familiarization with the same-texture columns, consistent with previously described aging WTD protocols (Mehdipour et al., 2019, 2021). After a 5-min resting interval, both columns were replaced, with one column containing the previously encountered texture and the other containing a novel texture. Mice were allowed to explore the familiar and novel textures during a 5-min testing phase. Behavioral sessions were recorded using an iPad Air (5th generation), and interaction time with the familiar and novel textures was quantified offline according to previously described WTD scoring criteria (Chen et al., 2018; Mehdipour et al., 2019, 2021) by an experimenter blinded to age group and morphometric results. The discrimination index was calculated as (novel texture interaction time − familiar texture interaction time)/(novel texture interaction time + familiar texture interaction time). Encoding index was calculated similarly as (left texture interaction time – right texture interaction time)/(left + right texture interaction time). Behavior-related analyses were restricted to animals with both WTD performance and corresponding morphometric data. Animals included in the behavior–morphometry analyses were euthanized within 24 h after WTD testing, an interval shorter than the 2-day imaging period over which prior *in vivo* imaging showed minimal net displacement of dendritic arbor tips in barrel cortex (Chen et al., 2018).

### 2.8 Composite DS and analysis of architecture–behavior coupling

Neuronal and microglial datasets were analyzed separately because they were obtained from different animal cohorts. For each cell type, a composite DS was generated by z-standardizing the Conventional Morphology DS and Spatial Architecture DS across animals within that cohort and averaging the two standardized scores:

Composite DS = [z(Conventional Morphology DS) + z(Spatial Architecture DS)]/2.

The WTD was introduced only after composite DS construction, when DS–WTD associations were examined. Composite DS was correlated with WTD discrimination index across all animals and separately within young and aged animals. Permutation analyses were used to test whether composite DS–WTD associations exceeded values expected under random assignment. For age-specific analyses, WTD values were permuted within each age group while composite DS values were held fixed, and Pearson’s r was recalculated for each permutation. For the age-specific analyses, the complete permutation spaces were exhaustively enumerated for both neuronal and microglial cohorts.

To test whether DS–WTD coupling differed directly between young and aged animals, the two independent age-specific Pearson correlations were compared using Fisher’s r-to-z transformation. The standardized difference between the Fisher-transformed correlations was calculated using the corresponding young and aged sample sizes. This direct comparison used the finalized composite DS values and did not reassign age labels.

For the within-age permutation analyses, two-tailed permutation *p*-values were calculated as the proportion of permuted correlation coefficients with absolute values greater than or equal to the corresponding observed correlation. The central 95% null interval was defined by the 2.5th and 97.5th percentiles of each corresponding null distribution.

### 2.9 Statistical analysis

All analyses were performed with the experimenter blinded to group identity whenever possible. GraphPad Prism 11 and Microsoft Excel 365 were used for data curation, general statistical analysis, PCA score handling, and data visualization. Permutation analyses and direct Fisher r-to-z comparisons were performed using custom scripts in Python 3. Animal number, reconstructed cell number, sex, and tissue sampling are provided in Supplementary Table 1.

Reconstructed-cell data were retained for morphometric description and visualization. For individual morphometric variables, age effects were evaluated using linear mixed-effects models with age as a fixed effect and animal identity as a random intercept. For Sholl and angular-distribution analyses, age, categorical distance or angular bin, and their interaction were included as fixed effects, with random intercepts for animal and reconstructed cell nested within animal. Young-aged contrasts at individual distances or angles were corrected using Šídák’s multiple-comparisons correction. Significance of fixed-effect terms was evaluated using likelihood-ratio tests comparing the full model with the corresponding reduced model.

Behavior-related analyses were performed at the animal level. Domain-level DS and RS associations with WTD discrimination index were evaluated using correlation analysis. Age-specific composite DS–WTD coupling was evaluated using the permutation procedures described in Section 2.8, whereas young–aged differences in correlation strength were tested using Fisher’s r-to-z comparison. Pearson correlation was used as the primary correlation measure. Spearman rank correlation was used when nonparametric sensitivity analysis was required. Correlation coefficients, 95% confidence intervals, and *p* values were reported whenever applicable. Statistical significance was set *a priori* at *p* < 0.05.

For an overview of the experimental and analytical workflow (see Figure 1A).

## 3 Results

### 3.1 Anatomical validation of L4 sampling and cellular reconstruction

We first established the anatomical and methodological basis for quantifying L4 cellular architecture in the primary somatosensory barrel field. Low-magnification images of Golgi-stained sections revealed the cortical region containing the barrel field and provided the anatomical context for selecting L4 pyramidal neurons for reconstruction (Figure 1B). Corresponding IBA1-stained sections showed the distribution of microglia across S1BF and demonstrated labeling suitable for analysis in anatomically matched L4 regions (Figure 1C).

To verify that reconstructed cells were sampled from the intended laminar position, soma depth was quantified from the pial surface to the center of the soma and plotted against the estimated L4 region (Figure 1D). This confirmed that reconstructed cells were located within the expected depth range of L4 barrel cortex. Higher-magnification images further illustrated the quality of cellular labeling used for reconstruction. Golgi staining provided clear visualization of pyramidal neuron somata and dendritic arbors (Figure 1E), whereas IBA1 immunohistochemistry labeled microglial somata and processes with sufficient contrast for process reconstruction and morphometric analysis (Figure 1F).

### 3.2 Aging reduces conventional dendritic morphometric features of L4 pyramidal neurons

We next examined whether normal aging altered the conventional morphology of L4 pyramidal neurons in S1BF. Representative Neurolucida reconstructions showed that young neurons generally exhibited more elaborate dendritic arbors, whereas aged neurons displayed a more restricted arborization pattern (Figure 2A). By contrast, somatic morphology was largely preserved with aging. Somatic variables did not differ significantly between young and aged neurons (Supplementary Figure 1). These findings suggested that aging may be associated primarily with dendritic, rather than somatic, remodeling in L4 pyramidal neurons.

**Figure 2:**
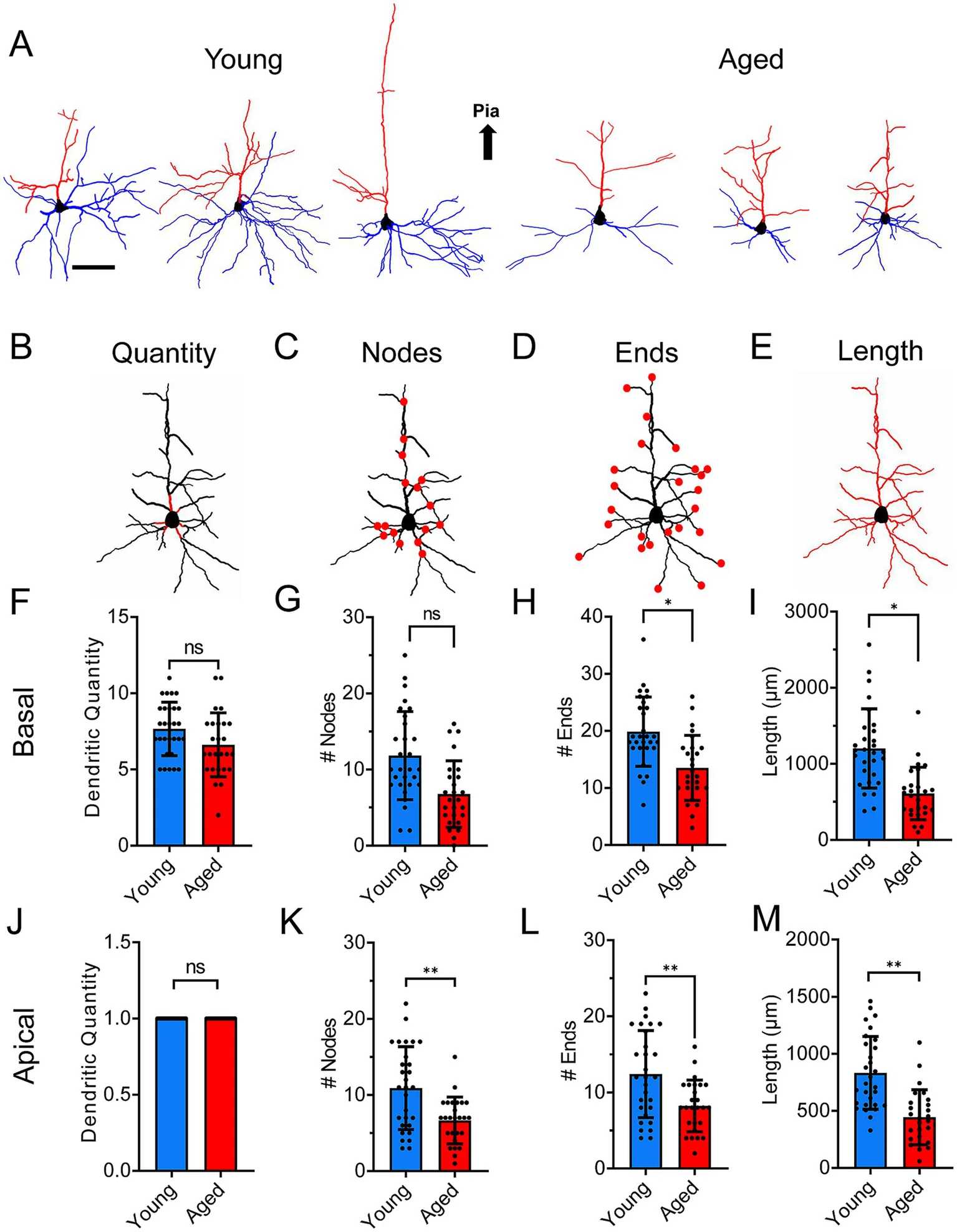
Aging reduces conventional dendritic morphometric features of L4 pyramidal neurons. **(A)** Representative Neurolucida reconstructions of L4 pyramidal neurons from young adult and aged mice. Apical dendrites are shown in red, basal dendrites in blue, and somata in black. **(B–E)** Schematics of conventional dendritic morphometric features in basal and apical dendritic compartments, including dendritic quantity **(B)**, dendritic nodes **(C)**, dendritic ends **(D)**, and total dendritic length **(E)**. Representative schematics above each column illustrate the corresponding morphometric feature. Aged neurons showed reduced dendritic arbor extent, with fewer dendritic ends **(H,L)** and shorter total dendritic length **(I,M)** in both compartments; dendritic nodes were reduced in the apical but not basal compartment **(G,K)**, whereas dendritic quantity was not significantly altered **(F,J)**. Each dot represents one reconstructed neuron. Bar graphs are shown as mean ± SD; ns, not significant; *p < 0.05, **p < 0.01. Scale bar: 50 μm in **(A)**.

To quantify this dendritic phenotype, we analyzed conventional morphometric measurements derived from reconstructed basal and apical dendritic compartments. Measurement schematics illustrate dendritic quantity, branch nodes, terminal ends, and cumulative dendritic length (Figures 2B–E). Dendritic quantity, defined as the number of primary dendritic trees emerging from the soma, was not significantly altered with aging. Basal dendritic quantity showed a slight, non-significant reduction in aged neurons (*p* = 0.0664), and apical dendritic quantity was unchanged across reconstructed cells (Figures 2F,J). Thus, age-related dendritic remodeling was not primarily expressed as a loss of primary dendritic trees.

Instead, the effects of aging appeared to emerge beyond these more proximal structural features and were expressed through alterations in the intermediate and distal organization of the dendritic arbor. Age-related changes in dendritic branching were compartment-specific. Dendritic nodes, defined as bi- or trifurcation branch points within the arbor, were not significantly altered in basal dendrites (*p* = 0.0768) but were reduced in apical dendrites (*p* = 0.0026; Figures 2G,K). Terminal dendritic ends were reduced with aging in both basal dendrites (*p* = 0.0234) and apical dendrites (*p* = 0.0046; Figures 2H,L). Cumulative dendritic length, which measures the total dendritic material contained within each compartment, showed the strongest age-related reduction. Aged neurons exhibited shorter basal dendritic length (*p* = 0.0169) and apical dendritic length (*p* = 0.0027; Figures 2I,M). Collectively, these findings suggested that aging preferentially affects the maintenance and retention of dendritic branches rather than the proximal scaffold from which they originate.

Together, our morphometric analyses revealed that normal aging reduces the conventional dendritic architecture of L4 pyramidal neurons in S1BF. This effect was expressed most consistently as fewer terminal dendritic endings and shorter cumulative dendritic length across both basal and apical compartments, while somatic morphology and primary dendritic quantity remained largely stable. These observations further suggested that age-related structural remodeling may be spatially patterned within the dendritic arbor. Complete nested mixed-effects model results for conventional neuronal morphometric variables are provided in Supplementary Table 2.

### 3.3 Aging reorganizes the spatial architecture of L4 pyramidal neuron dendrites

Because conventional morphometric analyses indicated that aging reduced dendritic branching and length while largely sparing somatic morphology and primary dendritic quantity, we next examined whether these changes were spatially organized within the dendritic arbor. Sholl analysis revealed that aging altered the radial distribution of dendritic material in both basal and apical compartments (Figures 3A–C). For basal dendrites, hierarchical mixed-effects analysis showed a significant age × distance interaction [*χ*^2^(29) = 367.91, *p* = 2.25 × 10^−60^], with Šídák-adjusted *post hoc* reductions from 40 to 110 μm from the soma (Figure 3B). Apical dendrites showed a corresponding age × distance interaction [*χ*^2^(41) = 237.21, *p* = 1.91 × 10^−29^], with reductions from 60 to 150 μm and at 170 μm (Figure 3C). Thus, aging reduced dendritic material primarily across intermediate zones of arbor elaboration.

**Figure 3:**
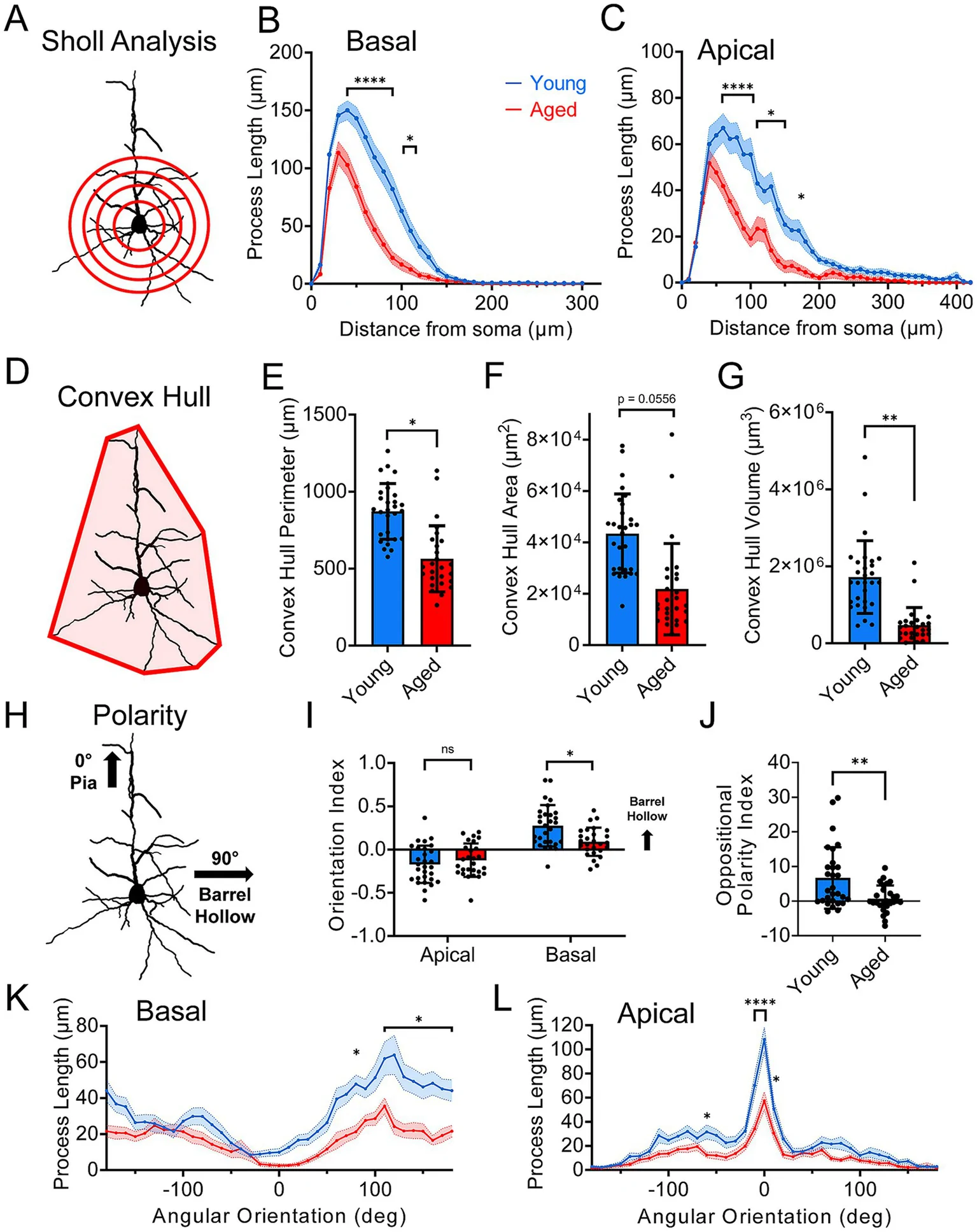
Aging reorganizes the spatial architecture and directional polarity of L4 pyramidal neuron dendrites. **(A)** Schematic illustrating Sholl analysis of dendritic process length as a function of distance from the soma. **(B,C)** Sholl analysis of basal **(B)** and apical **(C)** dendritic process length in young adult and aged L4 pyramidal neurons. Aging reduced dendritic process length across intermediate radial zones in both basal and apical dendritic compartments. **(D)** Schematic illustrating convex hull analysis used to quantify the spatial territory occupied by the dendritic arbor. **(E–G)** Convex hull perimeter **(E)** and volume **(G)** were reduced in aged neurons, whereas convex hull area **(F)** did not differ significantly after accounting for nesting. **(H)** Schematic illustrating the polarity coordinate system. The apical dendrite/pial direction was defined as 0°, and the barrel hollow-facing direction was defined as 90°. **(I)** Orientation index for apical and basal dendritic compartments. Positive values indicate greater dendritic orientation toward the barrel hollow. Apical orientation was not significantly altered with aging, whereas basal orientation toward the barrel hollow was reduced in aged neurons. **(J)** Oppositional polarity index showing reduced apical–basal dendritic polarity in aged neurons. **(K,L)** Angular distribution of basal **(K)** and apical **(L)** dendritic process length relative to the pia–barrel coordinate system. Aging reduced basal dendritic process length across barrel-facing angular sectors and reduced apical dendritic process length near the pia-oriented sector. Each dot represents one reconstructed neuron. For Sholl plots, horizontal brackets indicate radial intervals in which young–aged contrasts remained significant after Šídák correction; when adjacent bins are grouped under a single bracket, the symbol denotes the least stringent significance level shared across that interval. Bar graphs are shown as mean ± *SD*; Sholl and angular distribution plots are shown as mean ± SEM. ns, not significant; *p < 0.05, **p < 0.01, ***p < 0.001, ****p < 0.0001.

We next quantified the global dendritic territory occupied by each reconstructed neuron using convex hull analyses (Figure 3D). Aged L4 pyramidal neurons showed reduced convex hull perimeter (*p* = 0.0234) and volume (*p* = 0.0046), whereas convex hull area did not reach significance after accounting for nesting (*p* = 0.0556; Figures 3E–G). Together, these analyses showed that age-related dendritic remodeling was expressed not only as reduced branch complexity and length, but also as a spatial contraction of the dendritic field.

Although these analyses indicated that aging reduced radial dendritic distribution and overall dendritic territory, these measurements do not reveal the anatomical direction of dendritic remodeling. We therefore quantified dendritic fanning polarity relative to the geometry of L4 barrels, using an approach similar to that previously described (Chen et al., 2009). We operationally defined 0° as the direction of the apical dendrite toward the pia and 90° as the direction toward the estimated barrel center (Figure 3H). Apical and basal dendritic process lengths were quantified across angular bins to determine whether aging altered dendritic distribution relative to the pia–barrel axis.

Young L4 pyramidal neurons showed a clear compartment-specific polarity in their dendritic fanning: basal dendrites were preferentially oriented toward the barrel center, whereas apical dendrites were oriented away from it (Figures 3I,K,L). Apical orientation was not significantly altered with aging, whereas basal orientation toward the barrel center was reduced (*p* = 0.0405; Figure 3I). The oppositional polarity index was also reduced in aged neurons (*p* = 0.0065; Figure 3J).

Detailed analyses further identified the angular specificity of these age-related changes. Hierarchical mixed-effects analyses showed significant age × angular-orientation interactions for both apical dendrites [*χ*^2^(35) = 162.38, *p* = 2.58 × 10^−18^] and basal dendrites [*χ*^2^(35) = 131.02, *p* = 5.13 × 10^−13^]. Šídák-adjusted contrasts localized apical reductions to −60°, −10°, 0°, and +10° (Figure 3L), whereas basal reductions occurred at 80° and 110°–170° (Figure 3K). Together, the polarity analyses indicated that aging altered the directional organization of L4 pyramidal neuron dendrites relative to barrel geometry. Combined with the Sholl and convex hull results, these findings showed that normal aging impacts dendritic spatial architecture through radial contraction, reduced territorial occupancy, and weakened apical–basal polarity.

### 3.4 Aging reduces microglial process architecture and territorial occupancy in L4

We next examined whether normal aging altered microglial morphology in L4 of S1BF. Representative Neurolucida reconstructions of IBA1-labeled microglia showed that young microglia exhibited highly ramified process arbors, whereas aged microglia displayed a less elaborate process architecture (Figure 4A). To quantify this phenotype, we analyzed conventional microglial process measurements, including process quantity, branch nodes, terminal ends, and cumulative process length, all of which were significantly reduced after accounting for reconstructed cells nested within animals (all *p* < 0.01; Figures 4B–E). By contrast, whereas neuronal somatic variables were largely unchanged with aging, aged microglia exhibited pronounced somatic morphometric alterations, primarily reflecting enlarged cell bodies (Supplementary Table 3). Together, these findings indicated that aging altered conventional microglial morphology through enlarged somatic structure and reduced process elaboration across branching and length-based morphometric domains.

Because aging reduced conventional microglial process morphology, we next examined whether these changes were spatially organized around the soma. Sholl analysis revealed that aging altered the radial distribution of microglial process length (Figure 4F). Hierarchical mixed-effects analysis accounting for reconstructed cells nested within animals was used to evaluate the age-dependent radial distribution of microglial process length. Šídák-adjusted *post hoc* comparisons showed reduced process length in aged microglia from 10 to 30 μm from the soma (all adjusted *p* < 0.0001). Thus, aging reduced microglial process material across the main radial zone occupied by each reconstructed cell.

**Figure 4:**
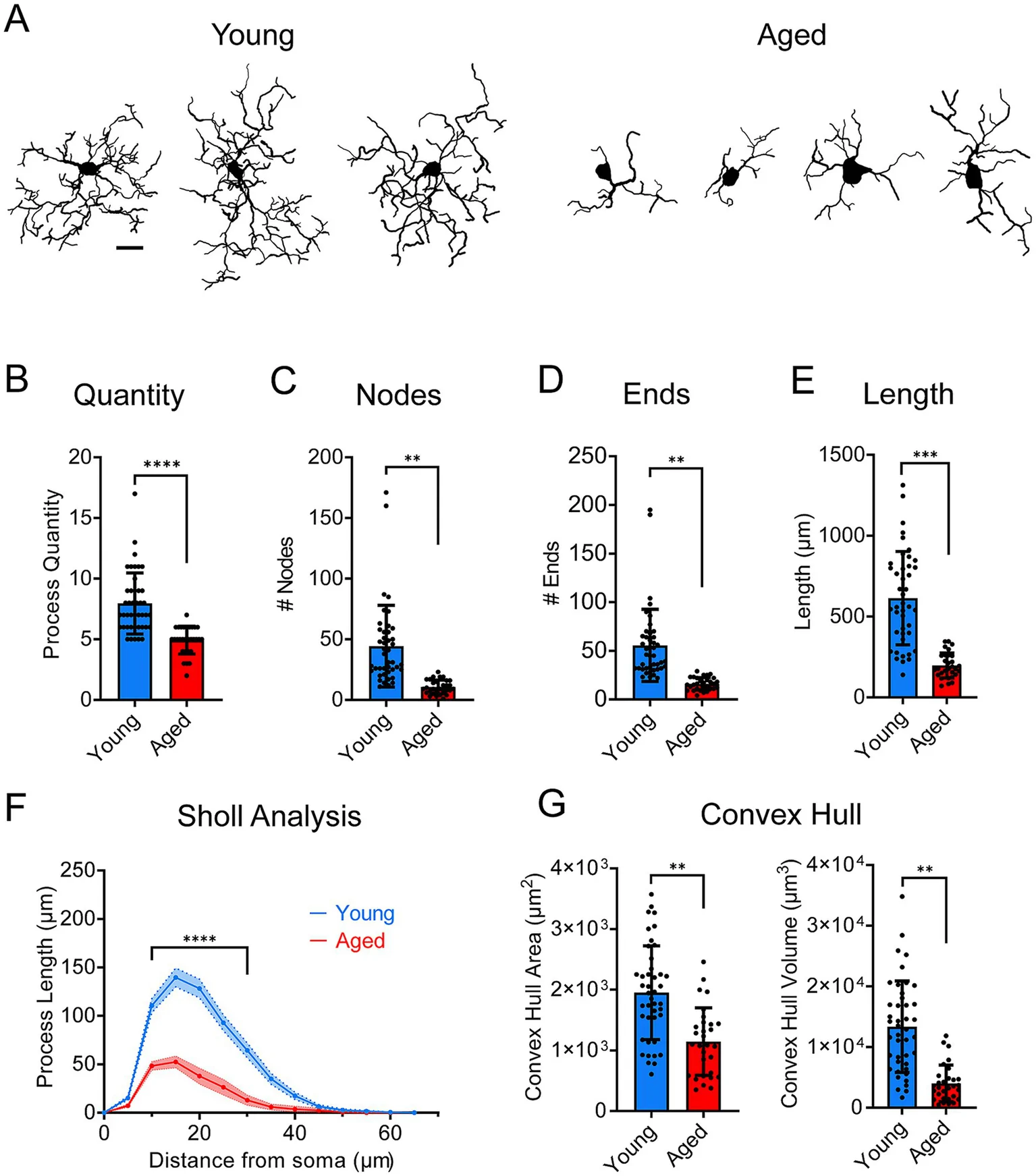
Aging reduces microglial process complexity and spatial coverage in L4 S1BF. **(A)** Representative Neurolucida reconstructions of IBA1-labeled microglia from young adult and aged mice. **(B–E)** Quantification of conventional microglial morphometric features, including process quantity **(B)**, nodes **(C)**, ends **(D)**, and total process length **(E)**. Aged microglia showed marked reductions in process quantity, branching complexity, terminal ends, and total process length compared with young adult microglia. **(F)** Sholl analysis of microglial process distribution as a function of distance from the soma. Aged microglia showed reduced process length across the proximal-to-intermediate arbor range. **(G)** Quantification of convex hull area and volume, showing reduced microglial spatial occupancy in aged mice. Each dot represents one reconstructed microglial cell. Bar graphs are shown as mean ± SD; Sholl analysis is shown as mean ± SEM. **p < 0.01; ***p < 0.001; ****p < 0.0001. Scale bar: 10 μm in **(A)**.

Finally, we quantified the territorial occupancy of each reconstructed microglial arbor using convex hull analyses. Aged microglia showed significantly reduced convex hull area (*p* = 0.0036) and volume (*p* = 0.0016; Figure 4G), indicating that aging constricted the spatial territory occupied by microglial processes. Together, these analyses showed that, in addition to L4 pyramidal neurons, microglial morphology is also impacted by normal aging, as expressed through fewer processes, reduced branching, shorter cumulative process length, restricted radial process distribution, and diminished territorial occupancy.

### 3.5 PCA-derived dominant architecture scores link L4 cellular architecture to whisker-dependent tactile performance

After identifying age-related changes in neuronal and microglial morphology, we next asked whether these diverse morphometric features could be distilled into dominant architectural signatures that relate to whisker-dependent tactile discrimination (WTD) performance. The WTD index measures the animal’s ability to discriminate a novel texture from a previously encountered texture using whisker-dependent tactile information. This behavioral readout therefore reflects sensory-guided tactile discrimination ability, which depends on the integrity of the barrel cortex (Chen et al., 2018). WTD performance has previously been shown to decline with aging (Mehdipour et al., 2019, 2021), a finding that was also replicated in the present study (Supplementary Figure 2). To test whether cellular architecture was associated with this behavioral capacity, we generated PCA-derived architecture scores for neuronal and microglial Conventional Morphology and Spatial Architecture domains. This approach allowed us to determine whether dominant morphological signatures were associated with the behavioral performance of individual animals.

In L4 pyramidal neurons, PCA identified dominant architecture components in both Conventional Morphology and Spatial Architecture domains. For Conventional Morphology, six retained PCs accounted for 92.1% of the total morphometric variance (Figure 5A), with loading patterns capturing coordinated variation across somatic, basal dendritic, and apical dendritic features (Figure 5B). For Spatial Architecture, three retained PCs accounted for 73.5% of the total variance (Figure 5D), with loadings reflecting contributions from convex hull features, Sholl-based radial distribution measures, proximal and distal arbor characteristics, and polarity indices (Figure 5E). When architecture scores were averaged at the animal level, neuronal Conventional Morphology DS was associated with WTD performance (*r* = 0.91, *p* < 0.001), whereas the corresponding RS showed a non-significant association (*r* = 0.43; Figure 5C). Neuronal Spatial Architecture DS was also associated with WTD performance (*r* = 0.92, *p* < 0.001), whereas Spatial Architecture RS was not significantly associated with WTD performance (*r* = 0.18; Figure 5F). In both neuronal domains, DS was associated with WTD performance, whereas the corresponding RS was not significantly associated with WTD.

**Figure 5:**
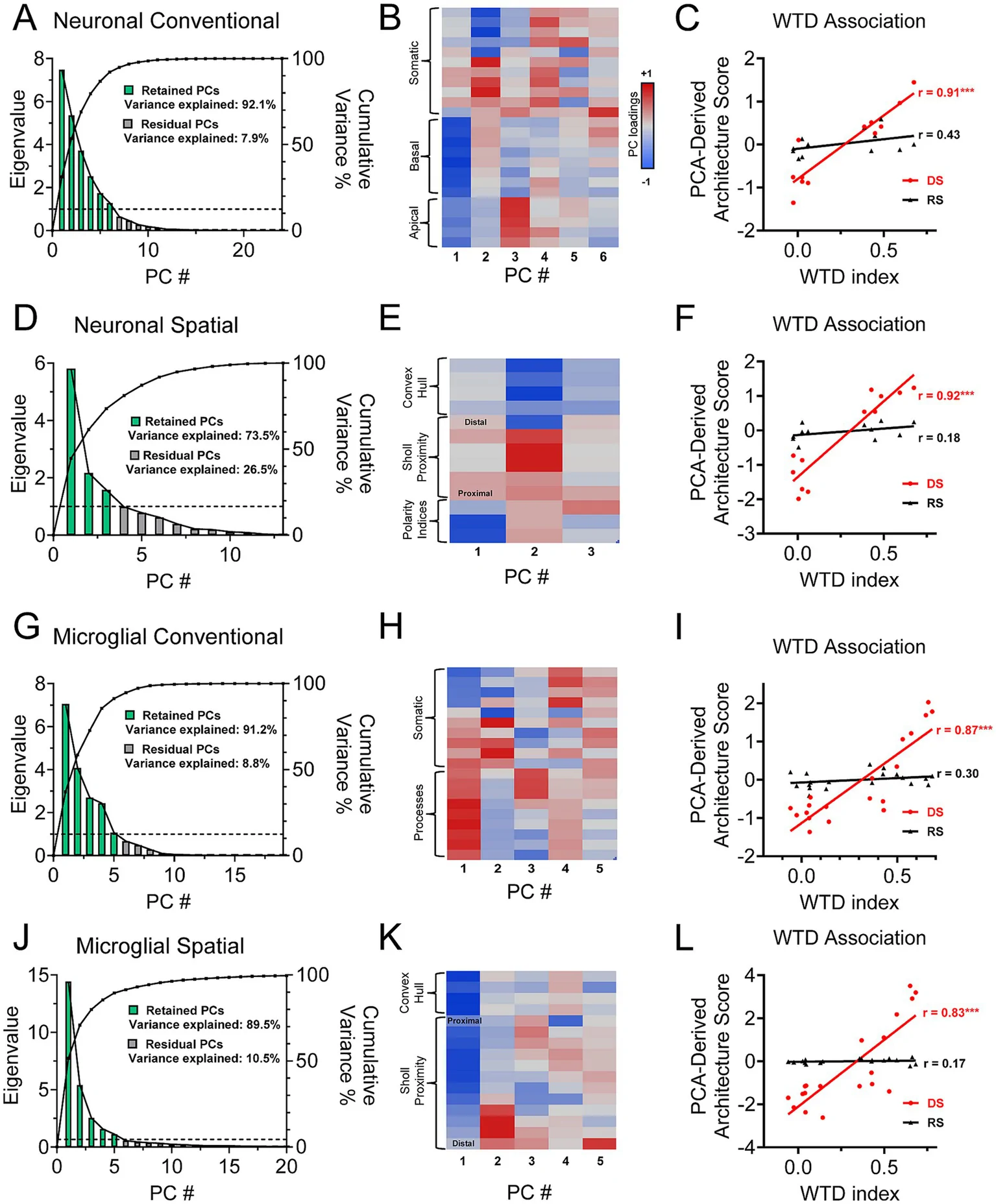
PCA-derived dominant architecture scores link L4 cellular morphometric organization to whisker-dependent tactile performance. **(A,D,G,J)** Scree plots showing eigenvalues and cumulative variance explained for PCA performed separately within each cellular morphometric domain. Green bars indicate retained PCs selected using the eigenvalue > 1 criterion, gray bars indicate non-retained PCs, and the black line shows cumulative variance explained. **(B,E,H,K)** Heatmaps showing loadings of retained PCs for neuronal conventional morphology **(B)**, neuronal spatial architecture **(E)**, microglial conventional morphology **(H)**, and microglial spatial architecture **(K)**. Variables are grouped according to their respective morphometric categories, and loading values are color-coded from −1 to +1. **(C,F,I,L)** Associations between PCA-derived architecture scores and whisker-dependent tactile discrimination (WTD) performance. Each dot represents data averaged for one animal. Dominant scores (DS), calculated from variance-weighted retained PCs, and residual scores (RS), calculated from variance-weighted non-retained PCs, were correlated with animal-level WTD performance. Across all four morphometric domains, DS showed positive associations with WTD performance, whereas RS showed non-significant associations. DS, dominant score; RS, residual score; WTD, whisker-dependent tactile discrimination. ***p < 0.001.

A comparable pattern was observed in reconstructed L4 microglia. For microglial Conventional Morphology, five retained PCs accounted for 91.2% of the total morphometric variance (Figure 5G), with retained PC loadings capturing coordinated variation across somatic and process-related microglial features (Figure 5H). For microglial Spatial Architecture, five retained PCs accounted for 89.5% of the total variance (Figure 5J), with loading patterns spanning convex hull measures and Sholl-based proximal and distal process distributions (Figure 5K). At the animal level, microglial Conventional Morphology DS was associated with WTD performance (*r* = 0.87, *p* < 0.001), whereas the corresponding RS showed a non-significant association (*r* = 0.30; Figure 5I). Microglial Spatial Architecture DS was also associated with WTD performance (*r* = 0.83, *p* < 0.001), whereas microglial Spatial Architecture RS was not significantly associated with WTD performance (*r* = 0.17; Figure 5L). Thus, across both neuronal and microglial datasets, WTD performance was consistently associated with DS, whereas the corresponding RS values showed non-significant associations.

For comparison with the WTD-related associations, DS values from each domain were also correlated with the encoding index. No significant pooled associations were observed across the four morphometric domains (Supplementary Figure 3). Together, these analyses showed that PCA-derived DS is associated with WTD behavior. Across both neuronal and microglial domains, higher DS values were consistently associated with better WTD performance, whereas RS values did not show such significant associations. These findings suggested that WTD performance is linked to dominant morphometric architecture derived from retained PCs, whereas the corresponding RS showed no significant relationship.

### 3.6 Aging weakens the coupling between L4 cellular architecture and whisker-dependent tactile discrimination

Having established a general pattern that PCA-derived dominant architecture signatures track WTD performance, we next asked whether this architecture–behavior relationship differs between young and aged animals. To address this question, we examined associations between composite DS values and WTD performance. These values integrated the Conventional Morphology DS and Spatial Architecture DS within each cellular dataset, providing a single measure of dominant cellular architecture. Because neuronal and microglial datasets were obtained from separate animal cohorts, the two cellular populations were evaluated independently. We then compared the strength of architecture–behavior coupling within each age group and assessed whether age-related differences exceeded those expected by chance.

In L4 pyramidal neurons, composite DS showed an age-dependent relationship with WTD performance (Figure 6A). Young animals showed a positive association between neuronal composite DS and WTD performance (*r* = 0.90, CI = 0.35–0.99, *p* < 0.05), indicating that animals with higher neuronal composite DS values showed better whisker-dependent tactile discrimination. By contrast, the corresponding association was not detected in aged animals (*r* = −0.38, CI = −0.91–0.63, *p* = 0.46). Permutation analysis confirmed this age-specific pattern. The observed young neuronal correlation exceeded the upper null threshold generated by within-age permutation (null threshold *r* = 0.84; Figure 6B), whereas the aged neuronal correlation fell within the corresponding null distribution (Figure 6C). A direct Fisher r-to-z comparison showed that neuronal DS–WTD coupling differed significantly between young and aged animals (*Z* = 2.32, *p* = 0.0203; Figure 6D), supporting an age-dependent weakening of neuronal architecture–behavior correspondence. Thus, L4 neuronal composite DS was associated with WTD performance in young animals.

**Figure 6:**
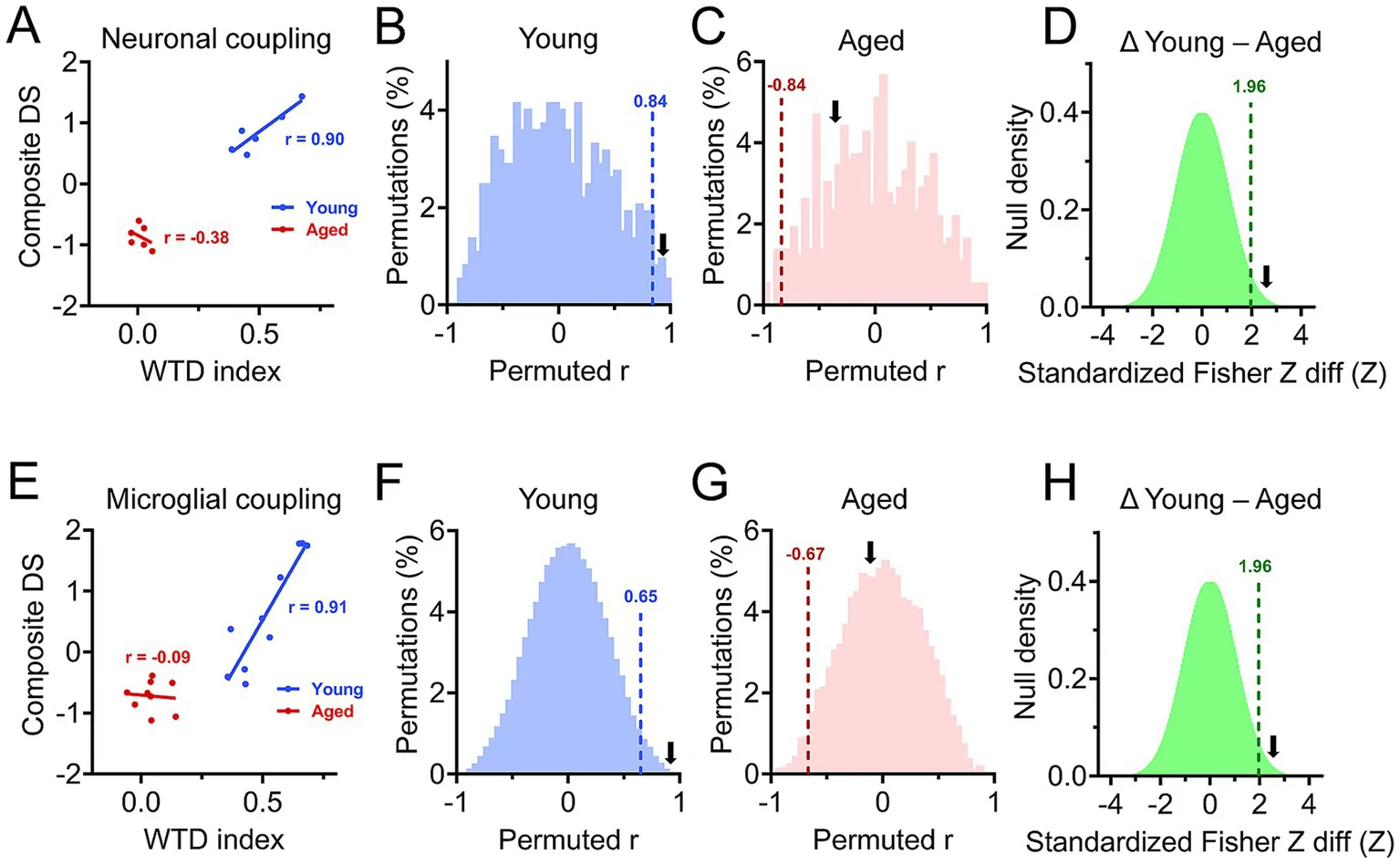
Aging weakens the coupling between L4 cellular architecture and whisker-dependent tactile discrimination. **(A–D)** Neuronal composite DS–WTD coupling analysis. **(A)** Relationship between neuronal composite DS and WTD discrimination index in young and aged animals. **(B,C)** Permutation null distributions for neuronal DS–WTD correlations in young **(B)** and aged **(C)** animals. **(D)** Standard normal null distribution for the direct Fisher r-to-z comparison of neuronal DS–WTD coupling between young and aged animals. Young animals showed significant neuronal DS–WTD coupling, whereas the aged-group association was not significant; the direct Fisher r-to-z comparison indicated a significant age-dependent difference in coupling. **(E–H)** Microglial composite DS–WTD coupling analysis. **(E)** Relationship between microglial composite DS and WTD discrimination index in young and aged animals. **(F,G)** Permutation null distributions for microglial DS–WTD correlations in young **(F)** and aged **(G)** animals. **(H)** Standard normal null distribution for the direct Fisher r-to-z comparison of microglial DS–WTD coupling between young and aged animals. As with neuronal DS, microglial DS was associated with WTD performance in young animals, whereas the aged-group association was not significant; the direct Fisher r-to-z comparison indicated a significant age-dependent difference in coupling. Black arrows indicate the observed test statistic. In panels **(B,C,F,G)**, dashed lines indicate the corresponding permutation-based significance threshold; in panels **(D,H)**, dashed lines indicate the critical value for the direct two-tailed Fisher r-to-z comparison.

A parallel analysis was performed for microglial architecture. Microglial composite DS showed a similar age-dependent pattern (Figure 6E). Young animals showed a positive association between microglial composite DS and WTD performance (*r* = 0.91, CI = 0.66–0.98, *p* < 0.001), whereas the corresponding association was not detected in aged animals (*r* = −0.09, CI = −0.71–0.61, *p* = 0.81). Permutation analysis again supported this age-specific organization. The observed young microglial correlation exceeded the upper null threshold generated by within-age permutation (null threshold r = 0.65; Figure 6F), whereas the aged microglial correlation fell within the null distribution (Figure 6G). A direct Fisher r-to-z comparison likewise showed that microglial DS–WTD coupling differed significantly between young and aged animals (*Z* = 2.92, *p* = 0.00355; Figure 6H). These findings indicated that microglial process architecture, like dendritic architecture, was associated with tactile discrimination performance in young animals, and this correspondence weakened in aged animals.

Overall, these findings revealed a common organizational principle across independent neuronal and microglial morphometric streams: in young animals, dominant L4 cellular architecture is associated with sensory-guided behavioral performance, whereas in aged animals this relationship is weakened. Importantly, the age-related decline was not limited to changes in cellular structure alone, but extended to the behavioral relevance of that structure. Thus, normal aging is associated with weakened architecture-behavior coupling across the two parallel cellular datasets.

## 4 Discussion

The present study shows that normal aging restricts L4 dendritic and microglial process architecture, alters the spatial organization of the local cellular architecture, and weakens the structure–behavior correspondence between morphometric signatures and behavioral performance. Across reconstructed neurons and microglia, aging was expressed through reduced cellular elaboration, restricted territorial occupancy, and altered spatial distribution. Dominant morphometric scores mapped onto WTD performance in young animals, whereas this correspondence was weaker in aged animals. Restricted cellular architecture and weakened architecture–behavior correspondence therefore emerge as complementary features of the aging sensory cortex. Cellular morphology remains biologically meaningful because it defines the spatial field through which neurons receive and integrate inputs, and through which microglia occupy, survey, and interact with the surrounding parenchyma (Häusser et al., 2000; London and Häusser, 2005; Nimmerjahn et al., 2005; Spruston, 2008; Tremblay et al., 2010; Cserép et al., 2020). By combining detailed reconstruction-based morphometry with PCA-derived architecture scores, the present study establishes a quantitative framework for examining how morphometric architecture within each cellular domain maps onto behavior and how this correspondence changes with age.

The whisker-to-barrel system provides an anatomically constrained setting for linking cellular structure to sensory-guided behavior. L4 barrel cortex contains discrete cellular aggregates corresponding to the mystacial vibrissae and serves as a principal thalamorecipient compartment for lemniscal whisker input from VPM thalamus (Woolsey and Van Der Loos, 1970; Petersen, 2007; Wimmer et al., 2010; Feldmeyer, 2012; Feldmeyer et al., 2013). Sensory experience shapes barrel cortex physiology, dendritic architecture, dendritic protrusions, spine dynamics, microglial morphology, extracellular matrix organization, and whisker-dependent behavior (Simons and Land, 1987; Carvell and Simons, 1996; Zuo et al., 2005; McRae et al., 2007; Yang et al., 2009; Chen et al., 2012, 2015; Kuhlman et al., 2014; Kalambogias et al., 2020; Chen and Brumberg, 2021). Texture discrimination in rodents depends on active whisking, tactile sampling, and cortical processing of texture-related information (von Heimendahl et al., 2007; Wu et al., 2013; Garion et al., 2014; Buetfering et al., 2022). WTD has also been used to relate barrel cortex synaptic and circuit alterations to sensory-guided behavioral performance (Chen et al., 2018) and to assess age-related sensory impairment and intervention effects (Mehdipour et al., 2019, 2021). Our present study places normal aging within this structure–function lineage by showing that age-sensitive L4 cellular architecture tracks WTD performance in young animals and loses this relationship in aged animals.

Our findings identify apical dendrite-bearing L4 pyramidal neurons as an aging-sensitive structural component of the barrel cortex. L4 excitatory neurons are morphologically heterogeneous, with spiny stellate neurons, star pyramidal neurons, and pyramidal neurons differing in dendritic configuration, axonal projection, and barrel-related input organization (Lübke et al., 2000; Lubke et al., 2003; Staiger et al., 2004; Egger et al., 2008; Feldmeyer, 2012; Young et al., 2023). Classic aging work in the mouse barrel cortex reported preserved dendritic length and segment number in L4 spiny stellate neurons across advanced age, suggesting that age-related dendritic atrophy is not uniform across all L4 excitatory populations (Coleman et al., 1986). The present findings extend this earlier work by identifying apical dendrite-bearing L4 pyramidal neurons as an aging-sensitive population in which dendritic geometry, territorial occupancy, and barrel-related spatial organization are altered with aging. These features are biologically coupled to dendritic function because arbor geometry constrains synaptic input availability, dendritic compartmentalization, electrotonic integration, and the spatial sampling of local circuit input (Häusser et al., 2000; London and Häusser, 2005; Spruston, 2008). Age-related reductions in dendritic material therefore narrow the spatial field available for synaptic input sampling and dendritic integration, reducing the cellular substrate through which L4 pyramidal neurons contribute to tactile cortical function.

The microglial findings extend the aging phenotype from dendritic input architecture to the glial domain. Microglial processes continuously survey the intact brain parenchyma and form dynamic contacts with neuronal somata, dendrites, spines, and synaptic compartments (Nimmerjahn et al., 2005; Tremblay et al., 2010; Cserép et al., 2020). Microglia regulate synaptic pruning, experience-dependent synapse remodeling, learning-related synapse formation, and activity-dependent circuit refinement (Tremblay et al., 2010; Schafer et al., 2012; Parkhurst et al., 2013; Badimon et al., 2020). Normal aging alters microglial morphology, inflammatory responsiveness, transcriptional state, and surveillance properties across brain regions and species (Harry, 2013; Menassa et al., 2022; Antignano et al., 2023; Godeanu et al., 2023; Li et al., 2023; Tieu et al., 2025). IBA1-based morphometry in the present study showed reduced process number, branching, terminal endings, cumulative process length, radial distribution, and convex hull territory in aged L4 microglia. These measurements provide anatomical evidence that age-related changes in microglial process architecture may be functionally relevant to how microglia occupy, survey, and participate in remodeling the local cortical environment. Molecular activation state, cytokine signaling, phagocytic activity, and disease-associated transcriptional identity remain outside the resolution and scope of morphology alone (Reddaway et al., 2023; Green and Rowe, 2024).

The microglia–behavior associations should therefore be interpreted within a structural-state framework. Microglial processes directly monitor synaptic compartments *in vivo*, and microglia–synapse interactions are shaped by sensory experience, indicating that microglial structure is sensitive to local synaptic and activity-related conditions (Wake et al., 2009; Tremblay et al., 2010). However, CSF1R-mediated microglial depletion can remove most microglia without necessarily producing broad impairment of baseline behavioral performance in wild-type mice, indicating that microglia may not be required for moment-to-moment behavioral execution (Elmore et al., 2014; Spangenberg et al., 2019). Conversely, microglial perturbation can impair learning-dependent synapse formation, and microglial replacement in the aged brain has been reported to reverse cognitive, synaptic, and neuronal deficits, supporting a role for microglia in long-timescale plasticity, maintenance, and recovery of neuronal circuit structure (Parkhurst et al., 2013; Elmore et al., 2018). Thus, microglial morphology is best treated here as a structural readout of the local cortical state and as a possible indicator of long-timescale circuit maintenance, rather than as evidence that microglia acutely execute tactile behavior.

The parallel neuronal and microglial findings place aging-related morphometric change at the level of the local L4 cellular environment. Dendritic arbors and microglial processes define complementary cellular territories for synaptic input organization, dendritic integration, parenchymal surveillance, and cellular interaction (Häusser et al., 2000; London and Häusser, 2005; Nimmerjahn et al., 2005; Tremblay et al., 2010; Cserép et al., 2020). Although the neuronal and microglial datasets were obtained from independent age-matched cohorts, both cellular types converged on the same aging-sensitive morphometric logic: dominant cellular organization tracked WTD performance across the age range, whereas this correspondence weakened within aged animals.

The PCA provided a quantitative approach for organizing this coordinated morphometric structure. PCA summarizes covariance among standardized variables into orthogonal components, allowing high-dimensional feature spaces to be represented by dominant axes of variation without using behavioral outcomes to define those axes (Jolliffe and Cadima, 2016). WTD performance did not enter component retention, PC orientation, DS/RS construction, or composite DS derivation. Dominant scores therefore represented coordinated cellular architecture rather than behavior-derived association scores. Across domains, DS values were associated with WTD performance, whereas the corresponding RS values were not significantly associated. These comparisons apply to the specific variance-weighted composite scores rather than the retained or residual PCA subspaces as a whole. Encoding-index analyses did not show significant pooled DS associations across the morphometric domains.

The age-specific coupling analyses further enhanced our understanding of the structure–behavior relationship in cortical aging. At the animal level, the age-dependent difference of DS–WTD correspondence indicates that dominant L4 cellular architecture was associated with tactile performance in young animals, whereas this correspondence becomes less informative of tactile performance with aging. Similar age-related changes in structure–function coupling have been described at larger systems scales, where anatomical connectivity constrains functional connectivity (Preti and Van De Ville, 2019) and aging alters the strength of that relationship across cognitive and vascular contexts (Zhang et al., 2024). Our present findings extend this principle to reconstructed cellular morphometrics by showing that L4 neuronal and microglial architecture retains behavioral correspondence in young animals and loses that correspondence with aging.

Several biological conditions may contribute to this loss of architecture–behavior coupling. Restricted WTD range in aged animals (vs. young, Brown–Forsythe *p* = 0.0037), particularly when accompanied by reduced architecture-score variance, may limit the extent to which the remaining architecture differences scale with WTD performance once cellular territories have shifted into a spatially constricted state. Aged tactile performance may also become increasingly constrained by synaptic loss, altered inhibitory balance, microglial molecular state, vascular dysfunction, metabolic stress, neuromodulatory decline, or impaired experience-dependent plasticity, all of which have been implicated in cortical or cognitive aging (Dickstein et al., 2007, 2013; Harry, 2013; McQuail et al., 2015; Mather and Harley, 2016; Camandola and Mattson, 2017; Toth et al., 2017; Voglewede et al., 2019; Menassa et al., 2022; Antignano et al., 2023; Li et al., 2023). The present study did not measure synaptic ultrastructure, electrophysiology, inflammatory signaling, vascular function, metabolism, or neuromodulatory tone. Our results remain in the realm of morphometry and correlational behavior: normal aging reduces L4 cellular architecture and weakens the relationship between dominant morphometric organization and tactile behavioral performance.

Several limitations define the interpretability of the present findings. The reconstructed neurons were identified by laminar position, Golgi morphology, and the presence of an apical dendrite, so the neuronal results apply to apical dendrite-bearing L4 pyramidal neurons rather than all genetically, projectionally, or electrophysiologically defined L4 excitatory subclasses (Lübke et al., 2000; Lubke et al., 2003; Staiger et al., 2004; Egger et al., 2008; Feldmeyer, 2012; Young et al., 2023). IBA1 reconstructions support conclusions about microglial process architecture and territory, while functional states such as inflammatory activation, phagocytosis, synaptic engulfment, and disease-associated transcriptional identity require molecular and functional validation (Reddaway et al., 2023; Green and Rowe, 2024). PCA-derived DS values are empirical summaries of coordinated morphometric variance, and larger datasets may be needed to test whether these architecture scores generalize across cohorts, cortical regions, interventions, behavioral tasks, or additional cellular types such as astrocytes and oligodendrocytes. The DS–WTD relationships identify architecture–behavior coupling, not causal sufficiency.

In conclusion, normal aging restricts the spatial occupancy of dendritic and microglial processes and weakens architecture-behavior correspondence in the sensory cortical circuit. The dominant morphometric signatures that track behavioral performance in young animals show weakened correspondence in aged animals, indicating that aging reduces the link between cellular organization and sensory function. These findings support a structural model of cortical aging in which parallel neuronal and microglial morphological changes provide an anatomical signature of aging-related decline in sensory-guided behavior.

## Data Availability

The original contributions presented in the study are included in the article/Supplementary material, further inquiries can be directed to the corresponding author.
