# Supplementary material for: Coordinated cellular architecture reveals weakened cortical structure–behavior coupling in normal aging
Source: Front Aging Neurosci. 2026 Sep 30;18:1932616. doi: 10.3389/fnagi.2026.1932616 (PMC13662101; doi:10.3389/fnagi.2026.1932616)
Supplement: Supplementary file 1 [file Data_Sheet_1.PDF]

Supplemental Material

Coordinated cellular architecture reveals weakened cortical structure-behavior coupling in normal aging

Xiaoyi Zhan, Pengcheng Tan, Tirzah Tan, and Chia-Chien Chen

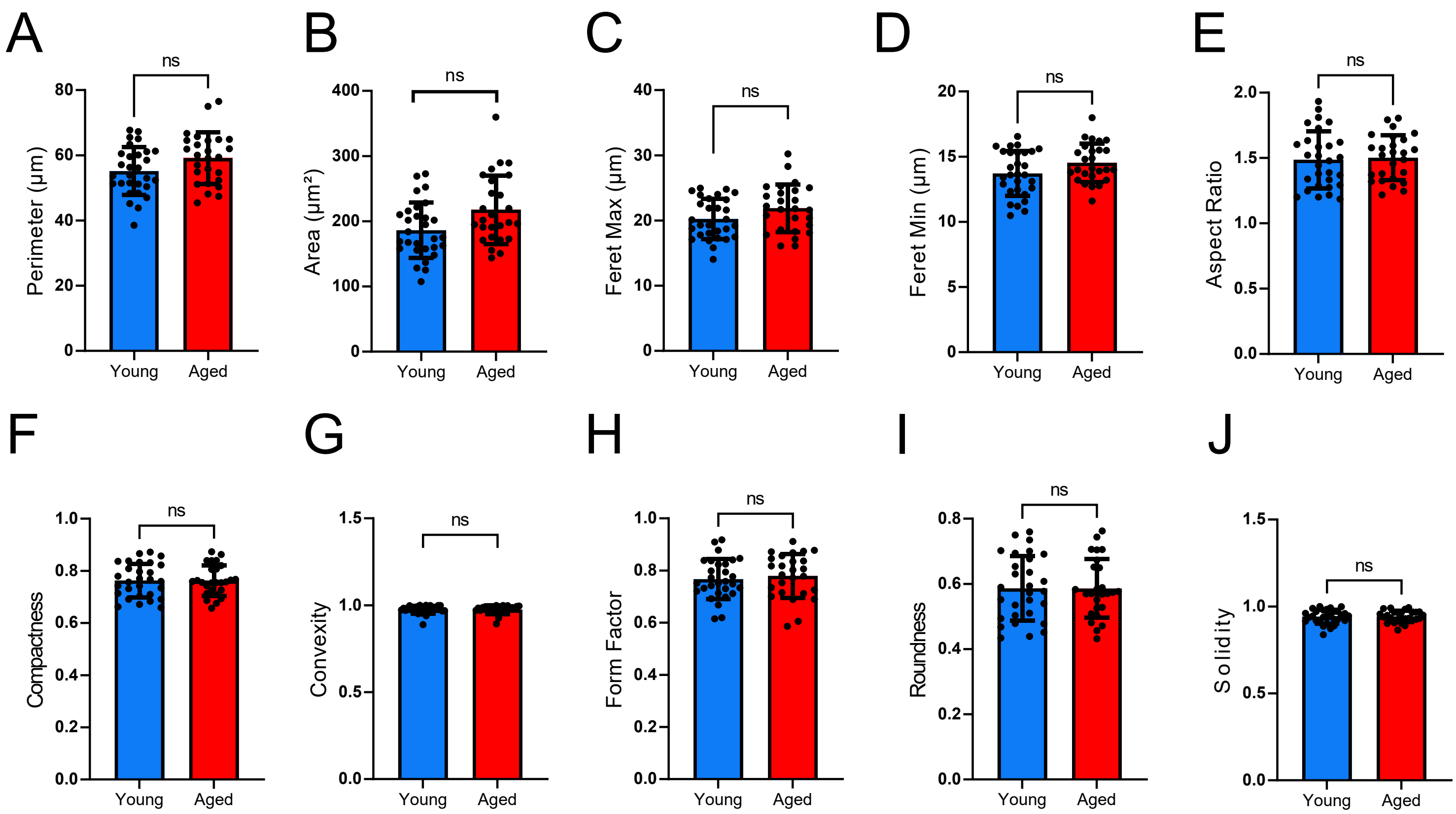

Supplemental Figure 1. L4 pyramidal neuron somatic morphology is largely preserved with aging. Somatic morphometric variables were quantified from Golgi-labeled L4 pyramidal neurons in young adult and aged mice. (A-J) Quantification of soma perimeter (A), soma area (B), maximum Feret diameter (C), minimum Feret diameter (D), aspect ratio (E), compactness (F), convexity (G), form factor (H), roundness (I), and solidity (J). No somatic morphometric variable differed significantly between young adult and aged mice after accounting for reconstructed cells nested within animals. Each dot represents one reconstructed neuronal soma. Bar graphs are shown as mean +/- SD. ns, not significant.

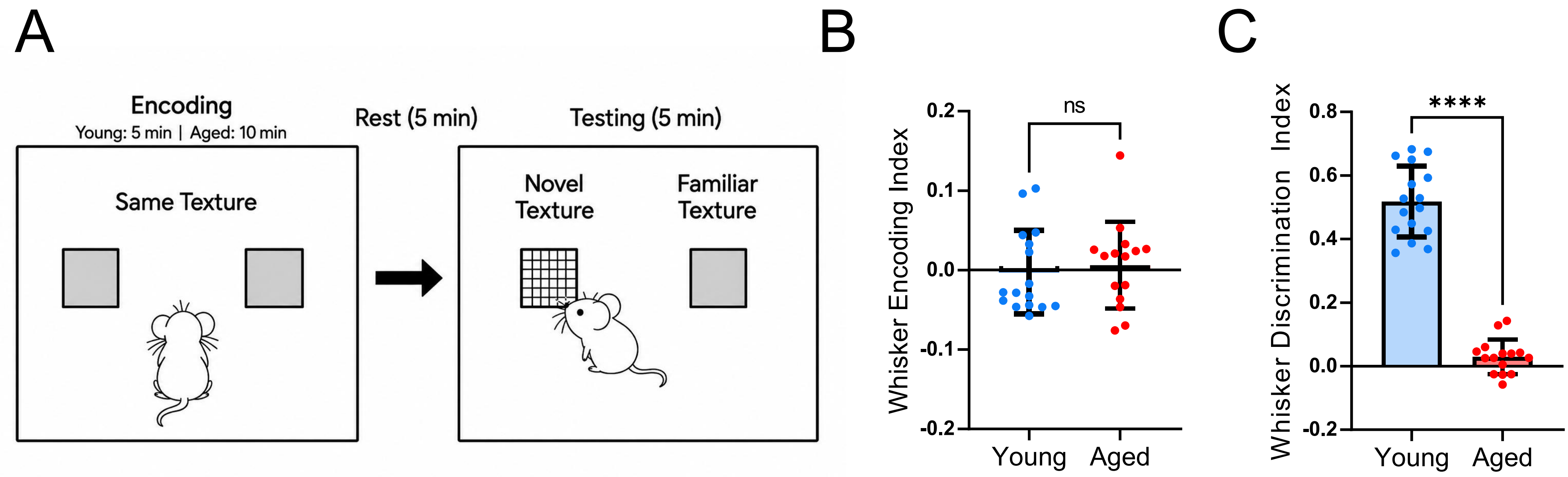

Supplemental Figure 2. Aging reduces whisker-dependent tactile discrimination without altering same-texture exploration bias. (A) Schematic of the whisker-dependent texture discrimination task. During the encoding phase, mice explored two columns consisting of the same texture. After a 5-min rest interval, mice were tested with one familiar texture and one novel texture. (B) Encoding index during the same-texture phase did not differ between young adult and aged mice, indicating no age-dependent side preference during initial exploration. (C) Whisker discrimination index was significantly reduced in aged mice compared with young adult mice. Each dot represents one animal. Bar graphs are shown as mean +/- SD. ns, not significant; ****$p < 0.0001$.

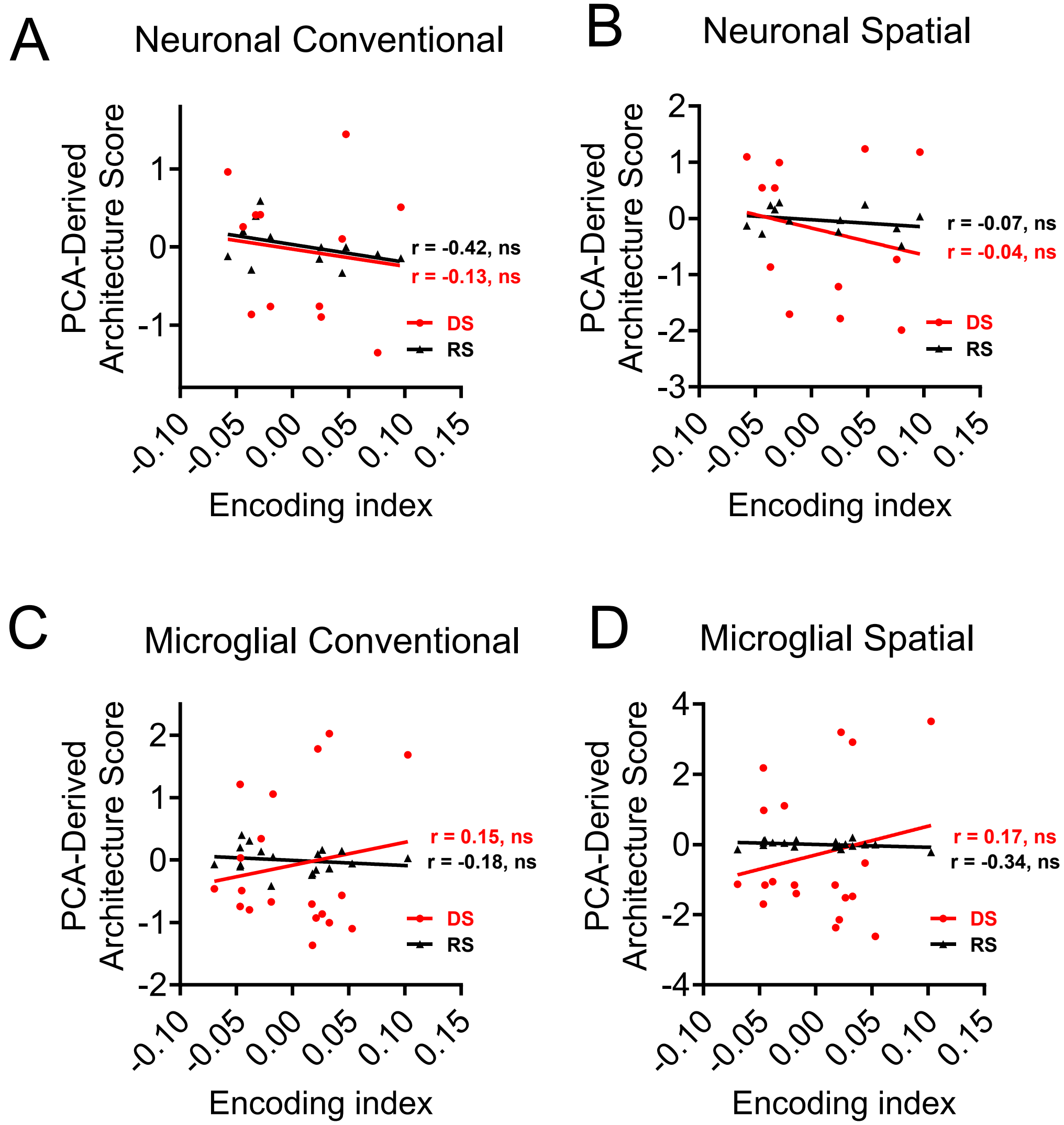

Supplemental Figure 3. PCA-derived architecture scores are not associated with encoding index. Associations between PCA-derived Dominant Scores (DS) and Residual Scores (RS) and encoding index are shown for neuronal conventional morphology (A), neuronal spatial architecture (B), microglial conventional morphology (C), and microglial spatial architecture (D). Each dot represents data averaged for one animal. No significant DS or RS associations with encoding index were observed across morphometric domains. DS, Dominant Score; RS, Residual Score. ns, not significant.

Supplemental Table 1. Sample composition and animal-level descriptive summaries for neuronal and microglial morphometric datasets.

The first section summarizes reconstructed-cell sampling by age group and sex. The subsequent sections provide animal-level descriptive summaries for the morphometric datasets underlying Figures 2–4. For each variable, reconstructed cells were first averaged within animal; values shown below are the mean ± SD of those animal-level means within each age-by-sex subgroup.

Cellular dataset	Age group	Animals (n)	Sex	Reconstructed cells	Mean cells / animal
**Golgi-labeled L4 pyramidal neurons**	Young adult	6	3 female, 3 male	29 neurons	4.8
	Aged	6	3 female, 3 male	26 neurons	4.3
**IBA1-labeled L4 microglia**	Young adult	10	5 female, 5 male	43 microglia	4.3
	Aged	9	5 female, 4 male	30 microglia	3.3

Note. Animal numbers for DS-WTD analyses correspond to animals with matched morphometric architecture scores and WTD behavioral data. Reconstructed-cell counts refer to the complete cell-level morphometric datasets used for descriptive morphometry and PCA. Neuronal and microglial cohorts were analyzed separately because they were obtained from separate tissue streams. The age-by-sex summaries below are descriptive only; no sex-stratified inferential tests or age-by-sex interaction tests were performed. Formal inference for age-related morphometric differences was based on the hierarchical mixed-effects analyses reported in the revised manuscript and statistical tables.

Part B. Animal-level descriptive summaries stratified by age and sex

Values are mean ± SD across animal-level means. Sex-stratified columns are provided descriptively to document sample composition and variability; they are not formal tests of sex effects or age-by-sex interactions.

Figure 2-related neuronal conventional morphometric variables

Domain	Measure	Young adult female (n = 3 animals)	Young adult male (n = 3 animals)	Aged female (n = 3 animals)	Aged male (n = 3 animals)
**Soma**	Perimeter (µm)	53.13 ± 6.36	57.94 ± 4.63	62.67 ± 6.83	59.33 ± 3.90
**Soma**	Area (µm²)	179.84 ± 31.71	203.57 ± 26.10	239.70 ± 63.29	220.85 ± 17.30
**Soma**	Feret max (µm)	19.38 ± 2.31	21.58 ± 2.04	23.09 ± 3.53	22.14 ± 1.40
**Soma**	Feret min (µm)	13.34 ± 1.00	14.05 ± 0.28	15.36 ± 1.30	14.52 ± 0.82
**Soma**	Aspect ratio	1.455 ± 0.073	1.552 ± 0.162	1.503 ± 0.116	1.517 ± 0.009
**Soma**	Compactness	0.775 ± 0.029	0.750 ± 0.027	0.758 ± 0.019	0.760 ± 0.026
**Soma**	Convexity	0.976 ± 0.021	0.976 ± 0.015	0.964 ± 0.030	0.982 ± 0.013
**Soma**	Form factor	0.789 ± 0.075	0.764 ± 0.029	0.762 ± 0.046	0.789 ± 0.069
**Soma**	Roundness	0.604 ± 0.046	0.567 ± 0.044	0.579 ± 0.033	0.580 ± 0.039
**Soma**	Solidity	0.959 ± 0.036	0.953 ± 0.029	0.941 ± 0.013	0.946 ± 0.036
**Basal dendrites**	Dendritic quantity	8.46 ± 0.56	7.64 ± 1.23	6.83 ± 1.26	6.62 ± 1.07
**Basal dendrites**	Nodes	10.85 ± 4.40	9.26 ± 3.48	8.89 ± 5.30	6.08 ± 0.66
**Basal dendrites**	Ends	19.60 ± 4.37	17.03 ± 2.66	15.85 ± 6.21	12.70 ± 1.58
**Basal dendrites**	Total length (µm)	1163.34 ± 425.80	993.97 ± 199.21	822.31 ± 452.37	568.86 ± 86.49
**Basal dendrites**	Mean length (µm)	145.12 ± 61.07	136.71 ± 43.62	119.35 ± 49.63	89.02 ± 20.63
**Basal dendrites**	Total surface (µm²)	5528.45 ± 1402.45	4998.62 ± 1048.43	3376.95 ± 1209.88	2612.85 ± 248.58
**Basal dendrites**	Mean surface (µm²)	693.04 ± 226.52	679.23 ± 137.31	507.73 ± 140.86	413.41 ± 37.67
**Basal dendrites**	Total volume (µm³)	2240.95 ± 518.78	2198.72 ± 725.86	1307.06 ± 642.22	1044.13 ± 160.43
**Basal dendrites**	Mean volume (µm³)	283.26 ± 89.20	293.71 ± 48.10	203.24 ± 100.52	168.43 ± 21.19
**Apical dendrites**	Dendritic quantity	1.00 ± 0.00	1.00 ± 0.00	1.00 ± 0.00	1.00 ± 0.00
**Apical dendrites**	Nodes	12.42 ± 1.63	10.49 ± 2.90	8.72 ± 3.86	6.48 ± 1.13
**Apical dendrites**	Ends	13.86 ± 1.55	12.10 ± 3.24	10.00 ± 3.61	8.59 ± 1.90
**Apical dendrites**	Total length (µm)	906.60 ± 134.95	895.09 ± 195.61	614.07 ± 343.84	462.81 ± 145.56
**Apical dendrites**	Surface (µm²)	4868.67 ± 1526.08	5398.99 ± 1698.76	2616.82 ± 1133.88	2616.08 ± 884.32
**Apical dendrites**	Volume (µm³)	3071.43 ± 151.47	3643.33 ± 330.24	1801.64 ± 855.95	1850.40 ± 208.12

Figure 3-related neuronal spatial architecture and polarity variables

The table includes convex-hull measures, all Sholl intersection and process-length radii, all angular dendritic-length bins, and polarity indices contained in the Figure 3 analysis datasets.

Domain	Measure	Young adult female (n = 3 animals)	Young adult male (n = 3 animals)	Aged female (n = 3 animals)	Aged male (n = 3 animals)
**Convex hull**	Area (µm²)	48684.14 ± 10154.72	42835.55 ± 4085.14	38342.41 ± 31700.96	20200.24 ± 8069.86
**Convex hull**	Perimeter (µm)	897.75 ± 164.09	904.56 ± 76.98	739.02 ± 336.64	574.31 ± 167.09
**Convex hull**	Volume (µm³)	1609696.52 ± 710771.85	1366572.46 ± 424267.40	850406.85 ± 869018.60	361291.43 ± 167656.52
**Convex hull**	Surface (µm²)	113850.19 ± 13338.95	100581.22 ± 8948.03	84483.44 ± 68067.12	40819.88 ± 8989.69
**Basal Sholl intersections**	10 µm radius	5.12 ± 1.76	3.72 ± 0.95	2.47 ± 1.61	3.32 ± 1.44
**Basal Sholl intersections**	20 µm radius	11.01 ± 0.99	9.19 ± 0.42	9.57 ± 2.32	7.90 ± 1.60
**Basal Sholl intersections**	30 µm radius	12.64 ± 0.13	11.19 ± 0.42	10.28 ± 1.75	7.83 ± 0.27
**Basal Sholl intersections**	40 µm radius	12.85 ± 1.45	10.57 ± 1.19	8.85 ± 2.76	7.44 ± 0.77
**Basal Sholl intersections**	50 µm radius	9.97 ± 2.92	9.98 ± 0.86	6.43 ± 2.77	6.19 ± 1.28
**Basal Sholl intersections**	60 µm radius	8.97 ± 4.14	8.37 ± 1.48	5.42 ± 2.67	4.43 ± 1.84
**Basal Sholl intersections**	70 µm radius	8.22 ± 4.14	7.11 ± 2.14	5.10 ± 3.42	3.22 ± 1.84
**Basal Sholl intersections**	80 µm radius	6.99 ± 3.63	6.29 ± 2.31	3.67 ± 3.06	2.37 ± 0.95
**Basal Sholl intersections**	90 µm radius	5.04 ± 3.11	4.96 ± 1.98	2.94 ± 2.80	1.71 ± 0.60
**Basal Sholl intersections**	100 µm radius	3.88 ± 2.43	3.53 ± 1.36	2.60 ± 3.05	1.24 ± 0.48
**Basal Sholl intersections**	110 µm radius	2.89 ± 2.17	2.53 ± 1.36	1.99 ± 2.26	0.48 ± 0.17
**Basal Sholl intersections**	120 µm radius	1.82 ± 1.40	1.39 ± 1.27	1.50 ± 1.80	0.27 ± 0.35
**Basal Sholl intersections**	130 µm radius	1.44 ± 1.08	1.04 ± 0.93	1.33 ± 1.53	0.22 ± 0.38
**Basal Sholl intersections**	140 µm radius	0.99 ± 0.60	0.50 ± 0.50	0.72 ± 0.75	0.11 ± 0.19
**Basal Sholl intersections**	150 µm radius	0.71 ± 0.75	0.47 ± 0.50	0.61 ± 0.79	0.00 ± 0.00
**Basal Sholl intersections**	160 µm radius	0.29 ± 0.26	0.43 ± 0.51	0.50 ± 0.87	0.00 ± 0.00
**Basal Sholl intersections**	170 µm radius	0.42 ± 0.52	0.26 ± 0.36	0.50 ± 0.87	0.00 ± 0.00
**Basal Sholl intersections**	180 µm radius	0.08 ± 0.14	0.00 ± 0.00	0.00 ± 0.00	0.00 ± 0.00
**Basal Sholl intersections**	190 µm radius	0.08 ± 0.14	0.00 ± 0.00	0.00 ± 0.00	0.00 ± 0.00
**Basal Sholl intersections**	200 µm radius	0.08 ± 0.14	0.00 ± 0.00	0.00 ± 0.00	0.00 ± 0.00
**Basal Sholl intersections**	210 µm radius	0.08 ± 0.14	0.00 ± 0.00	0.00 ± 0.00	0.00 ± 0.00
**Basal Sholl intersections**	220 µm radius	0.08 ± 0.14	0.00 ± 0.00	0.00 ± 0.00	0.00 ± 0.00
**Basal Sholl intersections**	230 µm radius	0.08 ± 0.14	0.00 ± 0.00	0.00 ± 0.00	0.00 ± 0.00
**Basal Sholl intersections**	240 µm radius	0.12 ± 0.22	0.00 ± 0.00	0.00 ± 0.00	0.00 ± 0.00
**Basal Sholl intersections**	250 µm radius	0.04 ± 0.07	0.00 ± 0.00	0.00 ± 0.00	0.00 ± 0.00
**Basal Sholl intersections**	260 µm radius	0.04 ± 0.07	0.00 ± 0.00	0.00 ± 0.00	0.00 ± 0.00
**Basal Sholl intersections**	270 µm radius	0.04 ± 0.07	0.00 ± 0.00	0.00 ± 0.00	0.00 ± 0.00
**Basal Sholl intersections**	280 µm radius	0.04 ± 0.07	0.00 ± 0.00	0.00 ± 0.00	0.00 ± 0.00
**Basal Sholl intersections**	290 µm radius	0.04 ± 0.07	0.00 ± 0.00	0.00 ± 0.00	0.00 ± 0.00
**Basal Sholl intersections**	300 µm radius	0.04 ± 0.07	0.00 ± 0.00	0.00 ± 0.00	0.00 ± 0.00
**Basal Sholl process length**	10 µm radius (µm)	34.85 ± 31.67	7.41 ± 4.96	5.10 ± 4.21	9.22 ± 5.26
**Basal Sholl process length**	20 µm radius (µm)	131.86 ± 5.67	95.23 ± 5.29	95.65 ± 35.50	75.45 ± 16.48
**Basal Sholl process length**	30 µm radius (µm)	147.67 ± 30.59	130.74 ± 8.15	134.88 ± 27.59	97.52 ± 16.68
**Basal Sholl process length**	40 µm radius (µm)	140.52 ± 40.91	139.13 ± 10.57	123.65 ± 31.56	92.77 ± 4.90
**Basal Sholl process length**	50 µm radius (µm)	122.41 ± 54.08	128.80 ± 21.56	97.27 ± 35.32	87.75 ± 11.68
**Basal Sholl process length**	60 µm radius (µm)	107.48 ± 65.59	110.22 ± 15.40	71.82 ± 40.13	66.81 ± 13.52
**Basal Sholl process length**	70 µm radius (µm)	94.41 ± 64.97	90.13 ± 27.11	60.75 ± 36.61	46.84 ± 20.94
**Basal Sholl process length**	80 µm radius (µm)	79.41 ± 68.02	78.18 ± 23.46	52.07 ± 33.71	32.04 ± 16.58
**Basal Sholl process length**	90 µm radius (µm)	64.06 ± 55.25	68.10 ± 25.29	38.96 ± 34.79	23.15 ± 9.72
**Basal Sholl process length**	100 µm radius (µm)	48.05 ± 45.29	49.53 ± 16.32	34.61 ± 42.22	16.03 ± 4.99
**Basal Sholl process length**	110 µm radius (µm)	34.74 ± 37.88	33.68 ± 16.85	28.58 ± 34.53	11.42 ± 6.04
**Basal Sholl process length**	120 µm radius (µm)	22.91 ± 26.54	22.74 ± 15.00	19.35 ± 23.25	4.61 ± 2.31
**Basal Sholl process length**	130 µm radius (µm)	13.89 ± 18.88	16.19 ± 15.85	19.12 ± 24.82	2.58 ± 3.74
**Basal Sholl process length**	140 µm radius (µm)	7.79 ± 10.61	8.38 ± 7.36	10.39 ± 10.59	2.37 ± 4.10
**Basal Sholl process length**	150 µm radius (µm)	4.64 ± 6.09	5.49 ± 5.71	8.60 ± 10.97	0.27 ± 0.46
**Basal Sholl process length**	160 µm radius (µm)	2.17 ± 3.76	4.84 ± 5.20	9.83 ± 14.51	0.00 ± 0.00
**Basal Sholl process length**	170 µm radius (µm)	1.26 ± 2.18	3.14 ± 3.80	7.35 ± 12.73	0.00 ± 0.00
**Basal Sholl process length**	180 µm radius (µm)	0.92 ± 1.60	2.06 ± 3.16	4.34 ± 7.51	0.00 ± 0.00
**Basal Sholl process length**	190 µm radius (µm)	0.97 ± 1.67	0.00 ± 0.00	0.00 ± 0.00	0.00 ± 0.00
**Basal Sholl process length**	200 µm radius (µm)	0.92 ± 1.59	0.00 ± 0.00	0.00 ± 0.00	0.00 ± 0.00
**Basal Sholl process length**	210 µm radius (µm)	1.02 ± 1.76	0.00 ± 0.00	0.00 ± 0.00	0.00 ± 0.00
**Basal Sholl process length**	220 µm radius (µm)	0.95 ± 1.65	0.00 ± 0.00	0.00 ± 0.00	0.00 ± 0.00
**Basal Sholl process length**	230 µm radius (µm)	0.98 ± 1.70	0.00 ± 0.00	0.00 ± 0.00	0.00 ± 0.00
**Basal Sholl process length**	240 µm radius (µm)	1.40 ± 2.43	0.00 ± 0.00	0.00 ± 0.00	0.00 ± 0.00

Domain	Measure	Young adult female (n = 3 animals)	Young adult male (n = 3 animals)	Aged female (n = 3 animals)	Aged male (n = 3 animals)
**Basal Sholl process length**	250 µm radius (µm)	0.76 ± 1.32	0.00 ± 0.00	0.00 ± 0.00	0.00 ± 0.00
**Basal Sholl process length**	260 µm radius (µm)	0.47 ± 0.81	0.00 ± 0.00	0.00 ± 0.00	0.00 ± 0.00
**Basal Sholl process length**	270 µm radius (µm)	0.47 ± 0.81	0.00 ± 0.00	0.00 ± 0.00	0.00 ± 0.00
**Basal Sholl process length**	280 µm radius (µm)	0.54 ± 0.93	0.00 ± 0.00	0.00 ± 0.00	0.00 ± 0.00
**Basal Sholl process length**	290 µm radius (µm)	0.46 ± 0.80	0.00 ± 0.00	0.00 ± 0.00	0.00 ± 0.00
**Basal Sholl process length**	300 µm radius (µm)	0.50 ± 0.87	0.00 ± 0.00	0.00 ± 0.00	0.00 ± 0.00
**Apical Sholl intersections**	10 µm radius	0.56 ± 0.51	0.31 ± 0.30	0.29 ± 0.51	0.51 ± 0.45
**Apical Sholl intersections**	20 µm radius	1.75 ± 0.25	1.74 ± 0.13	1.61 ± 0.35	1.73 ± 0.11
**Apical Sholl intersections**	30 µm radius	2.21 ± 0.83	3.16 ± 0.45	2.64 ± 0.92	2.62 ± 0.27
**Apical Sholl intersections**	40 µm radius	3.54 ± 1.39	3.28 ± 0.25	2.76 ± 0.32	3.62 ± 0.27
**Apical Sholl intersections**	50 µm radius	4.06 ± 1.42	4.00 ± 0.33	3.15 ± 0.46	3.17 ± 0.61
**Apical Sholl intersections**	60 µm radius	3.90 ± 1.29	4.81 ± 1.62	2.46 ± 0.56	3.05 ± 0.67
**Apical Sholl intersections**	70 µm radius	4.10 ± 0.89	4.91 ± 0.94	2.40 ± 0.81	2.44 ± 0.77
**Apical Sholl intersections**	80 µm radius	3.19 ± 1.13	4.97 ± 1.49	2.43 ± 0.28	1.54 ± 0.22
**Apical Sholl intersections**	90 µm radius	3.54 ± 1.37	4.64 ± 1.23	2.22 ± 0.63	1.03 ± 0.52
**Apical Sholl intersections**	100 µm radius	3.50 ± 1.32	4.23 ± 1.88	2.46 ± 1.37	1.22 ± 1.07
**Apical Sholl intersections**	110 µm radius	2.96 ± 1.00	3.47 ± 1.91	2.72 ± 1.25	1.56 ± 1.39
**Apical Sholl intersections**	120 µm radius	3.49 ± 1.75	3.13 ± 1.94	2.33 ± 1.53	1.00 ± 0.88
**Apical Sholl intersections**	130 µm radius	3.53 ± 1.54	2.56 ± 1.58	2.18 ± 2.11	0.67 ± 0.67
**Apical Sholl intersections**	140 µm radius	2.54 ± 1.25	2.31 ± 1.47	1.67 ± 2.08	0.44 ± 0.77
**Apical Sholl intersections**	150 µm radius	2.49 ± 1.44	2.13 ± 1.63	1.22 ± 1.58	0.56 ± 0.96
**Apical Sholl intersections**	160 µm radius	2.25 ± 1.64	1.80 ± 0.48	1.33 ± 1.53	0.56 ± 0.96
**Apical Sholl intersections**	170 µm radius	2.32 ± 1.69	1.73 ± 0.81	0.72 ± 0.75	0.56 ± 0.96
**Apical Sholl intersections**	180 µm radius	1.83 ± 1.26	1.03 ± 0.06	0.50 ± 0.87	0.44 ± 0.77
**Apical Sholl intersections**	190 µm radius	1.12 ± 0.82	0.97 ± 0.06	0.50 ± 0.87	0.22 ± 0.38
**Apical Sholl intersections**	200 µm radius	0.96 ± 0.90	0.97 ± 0.06	0.50 ± 0.87	0.22 ± 0.38
**Apical Sholl intersections**	210 µm radius	0.92 ± 0.95	0.68 ± 0.33	0.67 ± 1.15	0.33 ± 0.58
**Apical Sholl intersections**	220 µm radius	0.71 ± 1.12	0.68 ± 0.33	0.67 ± 1.15	0.33 ± 0.58
**Apical Sholl intersections**	230 µm radius	0.49 ± 0.74	0.68 ± 0.33	0.67 ± 1.15	0.22 ± 0.38
**Apical Sholl intersections**	240 µm radius	0.44 ± 0.77	0.58 ± 0.68	0.67 ± 1.15	0.11 ± 0.19
**Apical Sholl intersections**	250 µm radius	0.44 ± 0.77	0.58 ± 0.68	0.83 ± 1.44	0.11 ± 0.19
**Apical Sholl intersections**	260 µm radius	0.44 ± 0.77	0.76 ± 0.84	0.50 ± 0.87	0.11 ± 0.19
**Apical Sholl intersections**	270 µm radius	0.56 ± 0.96	0.47 ± 0.50	0.33 ± 0.58	0.11 ± 0.19
**Apical Sholl intersections**	280 µm radius	0.67 ± 1.15	0.43 ± 0.51	0.33 ± 0.58	0.11 ± 0.19
**Apical Sholl intersections**	290 µm radius	0.67 ± 1.15	0.43 ± 0.51	0.33 ± 0.58	0.11 ± 0.19
**Apical Sholl intersections**	300 µm radius	0.44 ± 0.77	0.37 ± 0.55	0.17 ± 0.29	0.11 ± 0.19
**Apical Sholl intersections**	310 µm radius	0.33 ± 0.58	0.48 ± 0.74	0.17 ± 0.29	0.11 ± 0.19
**Apical Sholl intersections**	320 µm radius	0.33 ± 0.58	0.48 ± 0.74	0.17 ± 0.29	0.00 ± 0.00
**Apical Sholl intersections**	330 µm radius	0.33 ± 0.58	0.48 ± 0.74	0.00 ± 0.00	0.00 ± 0.00
**Apical Sholl intersections**	340 µm radius	0.33 ± 0.58	0.37 ± 0.55	0.00 ± 0.00	0.00 ± 0.00
**Apical Sholl intersections**	350 µm radius	0.22 ± 0.38	0.37 ± 0.55	0.00 ± 0.00	0.00 ± 0.00
**Apical Sholl intersections**	360 µm radius	0.22 ± 0.38	0.26 ± 0.36	0.00 ± 0.00	0.00 ± 0.00
**Apical Sholl intersections**	370 µm radius	0.22 ± 0.38	0.14 ± 0.17	0.00 ± 0.00	0.00 ± 0.00
**Apical Sholl intersections**	380 µm radius	0.22 ± 0.38	0.14 ± 0.17	0.00 ± 0.00	0.00 ± 0.00
**Apical Sholl intersections**	390 µm radius	0.33 ± 0.58	0.14 ± 0.17	0.00 ± 0.00	0.00 ± 0.00
**Apical Sholl intersections**	400 µm radius	0.11 ± 0.19	0.03 ± 0.06	0.00 ± 0.00	0.00 ± 0.00
**Apical Sholl intersections**	410 µm radius	0.11 ± 0.19	0.00 ± 0.00	0.00 ± 0.00	0.00 ± 0.00
**Apical Sholl intersections**	420 µm radius	0.00 ± 0.00	0.00 ± 0.00	0.00 ± 0.00	0.00 ± 0.00
**Apical Sholl process length**	10 µm radius (µm)	1.17 ± 1.45	0.36 ± 0.47	0.49 ± 0.85	1.67 ± 1.59
**Apical Sholl process length**	20 µm radius (µm)	16.97 ± 2.44	13.32 ± 2.64	14.44 ± 6.50	17.92 ± 5.18
**Apical Sholl process length**	30 µm radius (µm)	29.64 ± 17.08	40.63 ± 3.33	34.88 ± 7.84	34.38 ± 4.16
**Apical Sholl process length**	40 µm radius (µm)	50.27 ± 32.53	52.16 ± 10.57	46.42 ± 18.41	55.31 ± 5.74
**Apical Sholl process length**	50 µm radius (µm)	62.67 ± 25.77	52.95 ± 10.15	46.63 ± 4.35	47.98 ± 8.02
**Apical Sholl process length**	60 µm radius (µm)	58.63 ± 16.79	65.99 ± 12.51	42.29 ± 14.70	45.11 ± 13.29
**Apical Sholl process length**	70 µm radius (µm)	54.06 ± 12.20	68.33 ± 11.03	40.72 ± 13.65	36.85 ± 6.64
**Apical Sholl process length**	80 µm radius (µm)	56.35 ± 19.85	71.58 ± 27.13	40.04 ± 10.21	24.00 ± 5.62
**Apical Sholl process length**	90 µm radius (µm)	47.69 ± 10.81	66.30 ± 15.37	35.31 ± 11.40	20.73 ± 15.21
**Apical Sholl process length**	100 µm radius (µm)	55.87 ± 27.78	72.32 ± 32.34	32.32 ± 10.86	14.56 ± 9.35
**Apical Sholl process length**	110 µm radius (µm)	44.32 ± 19.14	54.08 ± 27.06	45.15 ± 26.03	18.99 ± 17.40
**Apical Sholl process length**	120 µm radius (µm)	42.72 ± 25.61	48.95 ± 38.11	39.48 ± 16.15	18.38 ± 18.99
**Apical Sholl process length**	130 µm radius (µm)	62.80 ± 35.70	35.56 ± 19.13	32.15 ± 24.83	9.26 ± 8.84
**Apical Sholl process length**	140 µm radius (µm)	43.60 ± 22.09	35.13 ± 24.58	26.40 ± 25.43	5.03 ± 8.12

Domain	Measure	Young adult female (n = 3 animals)	Young adult male (n = 3 animals)	Aged female (n = 3 animals)	Aged male (n = 3 animals)
**Apical Sholl process length**	150 µm radius (µm)	35.66 ± 20.86	30.40 ± 24.49	17.64 ± 22.34	6.73 ± 11.66
**Apical Sholl process length**	160 µm radius (µm)	33.33 ± 24.05	25.78 ± 15.19	17.51 ± 21.33	7.99 ± 13.85
**Apical Sholl process length**	170 µm radius (µm)	31.72 ± 25.16	26.93 ± 11.03	12.06 ± 12.77	7.57 ± 13.10
**Apical Sholl process length**	180 µm radius (µm)	33.15 ± 24.48	14.95 ± 2.96	7.46 ± 9.90	7.59 ± 13.15
**Apical Sholl process length**	190 µm radius (µm)	22.72 ± 19.27	11.18 ± 1.17	6.03 ± 10.44	4.67 ± 8.09
**Apical Sholl process length**	200 µm radius (µm)	11.49 ± 10.79	10.73 ± 0.61	5.60 ± 9.69	2.25 ± 3.90
**Apical Sholl process length**	210 µm radius (µm)	10.79 ± 10.70	9.13 ± 1.25	5.82 ± 10.09	6.02 ± 10.43
**Apical Sholl process length**	220 µm radius (µm)	11.13 ± 12.06	7.00 ± 3.35	9.15 ± 15.84	6.12 ± 10.60
**Apical Sholl process length**	230 µm radius (µm)	7.76 ± 12.30	7.23 ± 3.65	9.92 ± 17.18	3.71 ± 6.43
**Apical Sholl process length**	240 µm radius (µm)	6.03 ± 10.16	7.14 ± 5.26	7.18 ± 12.44	1.46 ± 2.52
**Apical Sholl process length**	250 µm radius (µm)	4.83 ± 8.37	6.59 ± 6.78	8.78 ± 15.21	1.11 ± 1.93
**Apical Sholl process length**	260 µm radius (µm)	4.72 ± 8.18	8.52 ± 9.45	8.53 ± 14.78	1.20 ± 2.08
**Apical Sholl process length**	270 µm radius (µm)	4.86 ± 8.42	6.45 ± 7.31	5.29 ± 9.16	1.25 ± 2.17
**Apical Sholl process length**	280 µm radius (µm)	6.82 ± 11.81	4.47 ± 5.14	4.21 ± 7.29	1.13 ± 1.96
**Apical Sholl process length**	290 µm radius (µm)	7.58 ± 13.13	4.44 ± 5.13	4.93 ± 8.54	1.11 ± 1.93
**Apical Sholl process length**	300 µm radius (µm)	7.18 ± 12.44	4.20 ± 5.26	1.93 ± 3.33	1.14 ± 1.98
**Apical Sholl process length**	310 µm radius (µm)	4.68 ± 8.11	4.90 ± 7.53	1.95 ± 3.38	1.19 ± 2.06
**Apical Sholl process length**	320 µm radius (µm)	4.26 ± 7.38	4.94 ± 7.68	2.51 ± 4.35	0.99 ± 1.72
**Apical Sholl process length**	330 µm radius (µm)	3.72 ± 6.44	4.99 ± 7.74	0.86 ± 1.49	0.00 ± 0.00
**Apical Sholl process length**	340 µm radius (µm)	3.68 ± 6.37	4.09 ± 6.21	0.00 ± 0.00	0.00 ± 0.00
**Apical Sholl process length**	350 µm radius (µm)	3.97 ± 6.88	3.83 ± 5.76	0.00 ± 0.00	0.00 ± 0.00
**Apical Sholl process length**	360 µm radius (µm)	2.28 ± 3.95	3.22 ± 4.69	0.00 ± 0.00	0.00 ± 0.00
**Apical Sholl process length**	370 µm radius (µm)	2.25 ± 3.89	1.88 ± 2.41	0.00 ± 0.00	0.00 ± 0.00
**Apical Sholl process length**	380 µm radius (µm)	2.26 ± 3.91	1.46 ± 1.71	0.00 ± 0.00	0.00 ± 0.00
**Apical Sholl process length**	390 µm radius (µm)	4.09 ± 7.09	1.47 ± 1.74	0.00 ± 0.00	0.00 ± 0.00
**Apical Sholl process length**	400 µm radius (µm)	6.51 ± 11.28	1.31 ± 1.47	0.00 ± 0.00	0.00 ± 0.00
**Apical Sholl process length**	410 µm radius (µm)	1.85 ± 3.20	0.01 ± 0.01	0.00 ± 0.00	0.00 ± 0.00
**Apical Sholl process length**	420 µm radius (µm)	0.50 ± 0.87	0.00 ± 0.00	0.00 ± 0.00	0.00 ± 0.00
**Apical angular dendritic length**	0° (µm)	116.88 ± 63.98	124.30 ± 59.95	79.57 ± 35.23	55.66 ± 36.98
**Apical angular dendritic length**	10° (µm)	52.51 ± 9.00	49.11 ± 22.15	43.76 ± 36.09	26.94 ± 20.31
**Apical angular dendritic length**	20° (µm)	30.04 ± 8.34	16.65 ± 7.96	23.13 ± 18.74	5.60 ± 5.14
**Apical angular dendritic length**	30° (µm)	10.33 ± 13.46	11.23 ± 6.89	18.22 ± 13.66	6.34 ± 5.65
**Apical angular dendritic length**	40° (µm)	14.31 ± 8.74	12.94 ± 3.36	16.60 ± 15.66	15.09 ± 1.38
**Apical angular dendritic length**	50° (µm)	18.26 ± 14.00	22.91 ± 15.66	12.39 ± 8.45	14.98 ± 7.98
**Apical angular dendritic length**	60° (µm)	25.06 ± 9.72	31.23 ± 23.22	19.61 ± 10.81	19.96 ± 16.20
**Apical angular dendritic length**	70° (µm)	33.18 ± 15.68	23.33 ± 16.83	10.30 ± 6.49	11.01 ± 6.98
**Apical angular dendritic length**	80° (µm)	43.61 ± 29.59	17.62 ± 4.37	8.51 ± 2.20	10.07 ± 6.91
**Apical angular dendritic length**	90° (µm)	29.99 ± 19.34	20.70 ± 10.88	9.83 ± 3.26	15.20 ± 10.94
**Apical angular dendritic length**	100° (µm)	12.84 ± 3.86	16.30 ± 8.54	10.32 ± 4.87	7.12 ± 2.57
**Apical angular dendritic length**	110° (µm)	14.69 ± 9.09	24.36 ± 17.90	9.57 ± 6.20	5.27 ± 0.89
**Apical angular dendritic length**	120° (µm)	14.56 ± 14.65	18.33 ± 22.95	8.38 ± 4.93	4.68 ± 2.28
**Apical angular dendritic length**	130° (µm)	13.00 ± 11.40	12.16 ± 11.55	9.68 ± 6.31	4.29 ± 0.51
**Apical angular dendritic length**	140° (µm)	4.00 ± 6.42	8.22 ± 4.98	5.50 ± 3.69	1.31 ± 0.10
**Apical angular dendritic length**	150° (µm)	7.85 ± 4.42	9.00 ± 9.99	5.22 ± 5.99	0.30 ± 0.52
**Apical angular dendritic length**	160° (µm)	4.59 ± 3.99	0.80 ± 0.90	5.68 ± 9.46	0.12 ± 0.21
**Apical angular dendritic length**	170° (µm)	2.17 ± 3.75	0.84 ± 1.19	8.72 ± 14.03	0.20 ± 0.34
**Apical angular dendritic length**	180° (µm)	2.44 ± 3.86	0.80 ± 1.38	3.98 ± 6.41	0.45 ± 0.40
**Apical angular dendritic length**	190° (µm)	2.03 ± 3.51	0.83 ± 0.77	4.14 ± 5.90	0.13 ± 0.23
**Apical angular dendritic length**	200° (µm)	2.18 ± 3.78	2.84 ± 2.70	5.68 ± 7.75	1.37 ± 1.05
**Apical angular dendritic length**	210° (µm)	8.52 ± 8.87	4.10 ± 4.93	8.15 ± 8.69	0.49 ± 0.44
**Apical angular dendritic length**	220° (µm)	16.07 ± 14.96	13.05 ± 5.49	8.13 ± 7.70	3.94 ± 1.83
**Apical angular dendritic length**	230° (µm)	12.05 ± 6.22	13.61 ± 9.49	17.36 ± 12.17	4.61 ± 4.62
**Apical angular dendritic length**	240° (µm)	12.93 ± 4.86	16.46 ± 10.56	15.61 ± 11.83	7.09 ± 4.87
**Apical angular dendritic length**	250° (µm)	24.28 ± 8.11	40.94 ± 35.85	19.10 ± 7.55	9.69 ± 1.21
**Apical angular dendritic length**	260° (µm)	16.33 ± 6.34	25.08 ± 11.04	17.18 ± 8.93	15.08 ± 7.76
**Apical angular dendritic length**	270° (µm)	16.11 ± 3.00	36.26 ± 11.12	19.20 ± 8.81	20.31 ± 5.16
**Apical angular dendritic length**	280° (µm)	37.57 ± 24.88	36.42 ± 10.31	16.97 ± 10.94	22.65 ± 7.91
**Apical angular dendritic length**	290° (µm)	34.07 ± 15.76	22.64 ± 6.70	22.62 ± 10.81	23.93 ± 9.18
**Apical angular dendritic length**	300° (µm)	41.10 ± 22.37	39.70 ± 20.22	17.05 ± 8.02	10.04 ± 2.09
**Apical angular dendritic length**	310° (µm)	27.32 ± 11.55	38.91 ± 19.34	21.58 ± 14.87	5.49 ± 5.33
**Apical angular dendritic length**	320° (µm)	32.84 ± 11.83	17.67 ± 2.94	19.21 ± 14.24	3.63 ± 4.09
**Apical angular dendritic length**	330° (µm)	27.88 ± 3.33	18.13 ± 13.70	25.85 ± 17.38	7.17 ± 3.44

Domain	Measure	Young adult female (n = 3 animals)	Young adult male (n = 3 animals)	Aged female (n = 3 animals)	Aged male (n = 3 animals)
**Apical angular dendritic length**	340° (µm)	28.24 ± 9.72	30.68 ± 28.77	29.20 ± 20.09	17.89 ± 9.51
**Apical angular dendritic length**	350° (µm)	68.79 ± 17.81	77.79 ± 34.79	41.23 ± 32.16	36.84 ± 23.37
**Basal angular dendritic length**	0° (µm)	7.86 ± 8.87	4.75 ± 6.73	5.23 ± 5.72	1.39 ± 0.26
**Basal angular dendritic length**	10° (µm)	10.48 ± 12.66	5.76 ± 7.25	3.57 ± 2.96	1.95 ± 1.01
**Basal angular dendritic length**	20° (µm)	15.12 ± 11.24	9.30 ± 9.42	3.36 ± 3.02	2.95 ± 0.49
**Basal angular dendritic length**	30° (µm)	18.92 ± 9.79	9.45 ± 8.72	5.78 ± 0.72	4.79 ± 3.03
**Basal angular dendritic length**	40° (µm)	16.55 ± 14.62	12.92 ± 15.96	8.57 ± 0.70	8.50 ± 5.35
**Basal angular dendritic length**	50° (µm)	17.18 ± 19.56	17.23 ± 17.90	10.06 ± 4.43	14.44 ± 3.92
**Basal angular dendritic length**	60° (µm)	33.69 ± 17.94	28.88 ± 23.20	23.49 ± 16.25	14.48 ± 4.58
**Basal angular dendritic length**	70° (µm)	35.29 ± 14.32	35.77 ± 17.35	16.14 ± 6.89	27.32 ± 20.30
**Basal angular dendritic length**	80° (µm)	37.82 ± 20.41	44.25 ± 7.76	25.45 ± 8.01	22.80 ± 12.78
**Basal angular dendritic length**	90° (µm)	38.13 ± 22.20	32.17 ± 20.01	28.50 ± 10.33	27.27 ± 11.15
**Basal angular dendritic length**	100° (µm)	52.30 ± 26.19	30.95 ± 22.20	30.71 ± 19.66	27.16 ± 19.67
**Basal angular dendritic length**	110° (µm)	61.55 ± 39.99	54.58 ± 27.65	42.68 ± 28.10	36.32 ± 2.42
**Basal angular dendritic length**	120° (µm)	58.72 ± 24.54	74.06 ± 72.28	36.05 ± 24.27	19.75 ± 8.38
**Basal angular dendritic length**	130° (µm)	47.74 ± 13.92	45.33 ± 21.35	32.04 ± 18.39	19.06 ± 3.75
**Basal angular dendritic length**	140° (µm)	46.79 ± 14.85	40.18 ± 16.96	27.85 ± 23.00	22.06 ± 11.22
**Basal angular dendritic length**	150° (µm)	42.30 ± 21.57	35.34 ± 13.17	33.05 ± 22.81	15.88 ± 10.32
**Basal angular dendritic length**	160° (µm)	48.09 ± 21.96	38.39 ± 3.79	20.05 ± 14.02	15.45 ± 4.39
**Basal angular dendritic length**	170° (µm)	50.46 ± 17.00	38.70 ± 10.14	29.88 ± 14.45	13.49 ± 4.07
**Basal angular dendritic length**	180° (µm)	44.78 ± 15.12	46.06 ± 16.08	32.14 ± 19.75	17.40 ± 3.46
**Basal angular dendritic length**	190° (µm)	46.53 ± 4.13	31.62 ± 10.57	27.89 ± 15.17	16.55 ± 7.56
**Basal angular dendritic length**	200° (µm)	41.48 ± 20.99	38.90 ± 16.82	29.70 ± 11.98	13.16 ± 7.22
**Basal angular dendritic length**	210° (µm)	27.55 ± 14.58	29.15 ± 8.13	18.08 ± 14.68	17.19 ± 4.73
**Basal angular dendritic length**	220° (µm)	28.35 ± 9.43	26.45 ± 5.15	21.14 ± 8.05	19.95 ± 10.03
**Basal angular dendritic length**	230° (µm)	24.46 ± 6.47	26.06 ± 20.16	32.98 ± 9.19	21.32 ± 13.84
**Basal angular dendritic length**	240° (µm)	27.52 ± 7.02	19.80 ± 3.85	25.74 ± 10.49	21.30 ± 6.25
**Basal angular dendritic length**	250° (µm)	19.06 ± 7.46	17.76 ± 7.32	25.47 ± 10.35	22.65 ± 13.10
**Basal angular dendritic length**	260° (µm)	20.82 ± 11.14	30.64 ± 11.87	26.63 ± 16.22	20.13 ± 11.64
**Basal angular dendritic length**	270° (µm)	38.86 ± 8.11	22.01 ± 16.36	25.92 ± 22.33	15.04 ± 1.06
**Basal angular dendritic length**	280° (µm)	27.61 ± 13.83	26.48 ± 0.66	23.26 ± 17.49	12.33 ± 4.55
**Basal angular dendritic length**	290° (µm)	20.84 ± 14.38	24.94 ± 10.73	16.74 ± 15.55	12.15 ± 3.87
**Basal angular dendritic length**	300° (µm)	24.54 ± 12.68	7.99 ± 9.77	14.83 ± 13.96	12.37 ± 6.32
**Basal angular dendritic length**	310° (µm)	13.59 ± 13.29	7.73 ± 8.06	13.76 ± 18.05	9.51 ± 8.09
**Basal angular dendritic length**	320° (µm)	12.21 ± 10.91	5.54 ± 4.13	23.93 ± 17.88	5.73 ± 3.43
**Basal angular dendritic length**	330° (µm)	11.93 ± 3.94	4.42 ± 2.41	13.37 ± 14.04	6.78 ± 1.59
**Basal angular dendritic length**	340° (µm)	8.28 ± 5.95	5.05 ± 4.85	4.85 ± 4.83	1.76 ± 2.37
**Basal angular dendritic length**	350° (µm)	8.35 ± 7.71	7.63 ± 0.39	5.34 ± 5.39	1.82 ± 1.90
**Polarity indices**	Apical orientation index	-0.112 ± 0.029	-0.209 ± 0.057	-0.152 ± 0.075	-0.122 ± 0.082
**Polarity indices**	Basal orientation index	0.207 ± 0.069	0.228 ± 0.163	0.066 ± 0.025	0.141 ± 0.179
**Polarity indices**	Oppositional polarity index	3.555 ± 3.031	6.756 ± 3.266	1.150 ± 1.498	0.553 ± 0.291

Figure 4-related microglial conventional and spatial morphometric variables

The table includes soma and process morphometrics, convex-hull measures, and all Sholl intersection and process-length radii contained in the Figure 4 analysis datasets.

Domain	Measure	Young adult female (n = 5 animals)	Young adult male (n = 5 animals)	Aged female (n = 5 animals)	Aged male (n = 4 animals)
**Microglial soma**	Perimeter (µm)	25.85 ± 2.80	28.06 ± 3.25	30.23 ± 2.85	29.34 ± 4.03
**Microglial soma**	Area (µm²)	35.10 ± 3.56	40.36 ± 3.65	46.73 ± 9.61	44.95 ± 11.51
**Microglial soma**	Feret max (µm)	9.22 ± 0.71	9.97 ± 0.96	11.22 ± 1.52	10.49 ± 1.31
**Microglial soma**	Feret min (µm)	5.70 ± 0.49	6.25 ± 0.30	6.81 ± 0.56	6.70 ± 1.40
**Microglial soma**	Aspect ratio	1.655 ± 0.062	1.632 ± 0.191	1.689 ± 0.212	1.620 ± 0.312
**Microglial soma**	Compactness	0.727 ± 0.022	0.729 ± 0.042	0.692 ± 0.049	0.719 ± 0.062
**Microglial soma**	Convexity	0.930 ± 0.037	0.930 ± 0.051	0.950 ± 0.017	0.955 ± 0.019
**Microglial soma**	Form factor	0.665 ± 0.083	0.661 ± 0.093	0.640 ± 0.054	0.659 ± 0.057
**Microglial soma**	Roundness	0.533 ± 0.031	0.539 ± 0.058	0.484 ± 0.068	0.523 ± 0.091
**Microglial soma**	Solidity	0.914 ± 0.043	0.910 ± 0.034	0.880 ± 0.034	0.881 ± 0.024
**Microglial processes**	Process quantity	8.17 ± 1.95	8.14 ± 1.61	4.96 ± 0.22	5.02 ± 0.80
**Microglial processes**	Nodes	52.82 ± 34.59	29.76 ± 14.49	10.59 ± 3.64	11.88 ± 5.18
**Microglial processes**	Ends	64.75 ± 39.36	39.90 ± 13.94	15.93 ± 4.26	17.23 ± 5.81
**Microglial processes**	Total process length (µm)	643.00 ± 241.21	487.03 ± 317.62	187.07 ± 39.05	215.49 ± 68.78
**Microglial processes**	Mean process length (µm)	79.59 ± 16.13	65.86 ± 46.65	37.74 ± 7.36	44.28 ± 11.86
**Microglial processes**	Total process surface (µm²)	951.43 ± 559.91	819.24 ± 680.85	410.26 ± 171.60	443.12 ± 80.10
**Microglial processes**	Mean process surface (µm²)	116.92 ± 53.32	110.10 ± 96.98	83.22 ± 34.54	94.97 ± 19.60
**Microglial processes**	Total process volume (µm³)	129.87 ± 95.48	121.47 ± 114.10	84.13 ± 49.23	80.98 ± 16.95
**Microglial processes**	Mean process volume (µm³)	15.97 ± 10.15	16.27 ± 16.11	17.16 ± 9.96	18.39 ± 6.32
**Convex hull**	Area (µm²)	2026.74 ± 409.02	1666.66 ± 824.58	1149.88 ± 343.84	1131.18 ± 377.15
**Convex hull**	Perimeter (µm)	176.06 ± 15.79	148.53 ± 34.95	131.23 ± 23.49	128.50 ± 23.25
**Convex hull**	Volume (µm³)	13937.25 ± 4736.42	10402.10 ± 8538.45	3605.43 ± 2034.55	4957.01 ± 2963.63
**Convex hull**	Surface (µm²)	4709.33 ± 973.66	3757.98 ± 1894.34	2446.36 ± 762.03	2497.39 ± 840.83
**Sholl intersections**	5 µm radius	6.71 ± 2.82	5.62 ± 1.37	4.49 ± 0.82	3.65 ± 1.04
**Sholl intersections**	10 µm radius	14.59 ± 3.20	13.24 ± 3.11	7.11 ± 1.04	8.08 ± 1.33
**Sholl intersections**	15 µm radius	15.79 ± 5.54	12.76 ± 4.83	5.95 ± 0.96	6.12 ± 1.55
**Sholl intersections**	20 µm radius	13.50 ± 3.66	9.72 ± 5.51	3.71 ± 2.49	4.85 ± 2.74
**Sholl intersections**	25 µm radius	9.44 ± 2.79	6.62 ± 5.99	2.21 ± 1.50	2.85 ± 1.93
**Sholl intersections**	30 µm radius	6.28 ± 3.14	4.14 ± 3.95	0.89 ± 0.93	1.35 ± 0.91
**Sholl intersections**	35 µm radius	2.74 ± 1.30	1.98 ± 2.16	0.52 ± 0.50	0.52 ± 0.44
**Sholl intersections**	40 µm radius	1.25 ± 1.16	0.88 ± 1.17	0.64 ± 0.59	0.08 ± 0.17
**Sholl intersections**	45 µm radius	0.27 ± 0.28	0.32 ± 0.52	0.23 ± 0.25	0.00 ± 0.00
**Sholl intersections**	50 µm radius	0.19 ± 0.32	0.32 ± 0.52	0.04 ± 0.09	0.00 ± 0.00
**Sholl intersections**	55 µm radius	0.05 ± 0.11	0.08 ± 0.11	0.00 ± 0.00	0.00 ± 0.00
**Sholl intersections**	60 µm radius	0.00 ± 0.00	0.04 ± 0.09	0.00 ± 0.00	0.00 ± 0.00
**Sholl process length**	5 µm radius (µm)	14.57 ± 10.93	13.35 ± 9.40	6.18 ± 3.67	6.94 ± 2.66
**Sholl process length**	10 µm radius (µm)	112.82 ± 33.02	98.22 ± 34.20	49.76 ± 6.12	47.25 ± 12.01
**Sholl process length**	15 µm radius (µm)	146.44 ± 53.08	116.61 ± 50.34	50.09 ± 6.23	57.80 ± 14.52
**Sholl process length**	20 µm radius (µm)	136.85 ± 51.82	102.50 ± 57.83	37.27 ± 15.33	43.69 ± 19.01
**Sholl process length**	25 µm radius (µm)	100.88 ± 36.22	72.81 ± 54.03	22.52 ± 14.80	34.32 ± 16.63
**Sholl process length**	30 µm radius (µm)	70.08 ± 33.92	47.36 ± 41.85	9.53 ± 7.77	16.87 ± 11.21
**Sholl process length**	35 µm radius (µm)	38.33 ± 18.66	25.82 ± 30.25	4.62 ± 4.84	6.04 ± 4.26
**Sholl process length**	40 µm radius (µm)	17.13 ± 13.89	13.97 ± 16.87	3.66 ± 3.48	2.17 ± 2.05
**Sholl process length**	45 µm radius (µm)	5.87 ± 5.16	5.64 ± 9.14	2.85 ± 2.68	0.40 ± 0.80
**Sholl process length**	50 µm radius (µm)	1.47 ± 2.45	3.24 ± 5.71	0.52 ± 0.56	0.00 ± 0.00
**Sholl process length**	55 µm radius (µm)	0.58 ± 1.29	2.20 ± 3.21	0.07 ± 0.15	0.00 ± 0.00
**Sholl process length**	60 µm radius (µm)	0.09 ± 0.20	0.62 ± 0.88	0.00 ± 0.00	0.00 ± 0.00

Note. For every variable in Part B, reconstructed cells were first averaged within each animal, and subgroup mean ± SD was then calculated across animals. Thus, these values are animal-level descriptive summaries rather than cell-level summaries. No p values are presented in Part B because inferential testing of age effects used hierarchical mixed-effects models that explicitly accounted for reconstructed cells nested within animal.

Supplemental Table 2. Nested mixed-effects model results for conventional neuronal morphometric variables.

Values are reported as reconstructed-cell mean and SD for young adult and aged L4 pyramidal neurons (29 young neurons from 6 animals; 26 aged neurons from 6 animals). Age effects were evaluated using linear mixed-effects models with age as a fixed effect and animal identity as a random intercept.

Morphometric variable	Young adult		Aged		Nested mixed-effects model		
	Mean	SD	Mean	SD	χ^2	df	p
**Soma**							
Soma perimeter (µm)	55.16	7.32	59.16	7.93	2.89	1	0.0892
Soma area (µm²)	186.30	42.50	217.79	52.47	3.30	1	0.0691
Feret maximum (µm)	20.23	3.07	21.88	3.65	2.40	1	0.1214
Feret minimum (µm)	13.71	1.73	14.53	1.48	3.54	1	0.0600
Aspect ratio	1.49	0.22	1.50	0.17	0.09	1	0.7614
Compactness	0.76	0.06	0.76	0.06	0.00	1	0.9819
Convexity	0.97	0.02	0.97	0.02	0.00	1	0.9523
Form factor	0.77	0.08	0.78	0.08	0.32	1	0.5726
Roundness	0.59	0.10	0.59	0.09	0.00	1	0.9938
Solidity	0.94	0.04	0.94	0.03	0.31	1	0.5761
**Basal dendrites**							
Dendritic quantity	7.66	1.76	6.62	2.10	3.37	1	0.0664
Dendritic nodes	11.83	5.79	6.77	4.38	3.13	1	0.0768
Dendritic ends	19.86	6.06	13.50	5.69	5.14	1	0.0234
Total dendritic length (µm)	1201.59	520.22	609.29	345.56	5.71	1	0.0169
Mean dendritic length (µm)	165.09	80.93	92.31	44.71	3.85	1	0.0497
Total dendritic surface area (µm²)	5488.66	2169.53	2732.40	1511.70	11.07	1	8.76×10^{-4}
Mean dendritic surface area (µm²)	755.94	352.56	420.48	230.32	8.49	1	0.0036
Total dendritic volume (µm³)	2146.92	971.27	1088.46	793.80	10.35	1	0.0013
Mean dendritic volume (µm³)	295.66	149.65	170.49	133.09	8.22	1	0.0041
**Apical dendrite**							
Dendritic quantity	1.00	0.00	1.00	0.00	—	—	—
Dendritic nodes	10.90	5.45	6.65	3.07	9.07	1	0.0026
Dendritic ends	12.41	5.72	8.23	3.39	8.03	1	0.0046
Total dendritic length (µm)	832.81	319.00	443.87	240.18	9.01	1	0.0027
Total dendritic surface area (µm²)	4393.84	2081.99	2352.02	1151.77	8.71	1	0.0032
Total dendritic volume (µm³)	3389.20	1276.24	1851.43	835.48	17.19	1	3.39×10^{-5}

Note. Likelihood-ratio statistics compare the random-intercept mixed model containing age with the corresponding model without the age term (df = 1). For apical dendritic quantity, all measured values were 1.00 in both groups (SD = 0.00), so no mixed-effects model was fitted. Numerical p values < 0.001 are shown in scientific notation.

Supplemental Table 3. Nested mixed-effects model results for conventional microglial morphometric variables.

Values are reported as reconstructed-cell mean and SD for young adult and aged IBA1-labeled microglia reconstructed from L4 S1BF (43 young microglia from 10 animals; 30 aged microglia from 9 animals). Age effects were evaluated using linear mixed-effects models with age as a fixed effect and animal identity as a random intercept.

Morphometric variable	Young adult		Aged		Nested mixed-effects model		
	Mean	SD	Mean	SD	χ^2	df	p
**Soma**							
Soma perimeter (µm)	27.01	4.71	29.86	4.93	3.48	1	0.0621
Soma area (µm²)	37.56	8.88	45.90	11.82	6.19	1	0.0128
Feret maximum (µm)	9.65	1.68	10.92	1.82	6.05	1	0.0139
Feret minimum (µm)	5.93	1.07	6.73	1.38	5.36	1	0.0207
Aspect ratio	1.67	0.38	1.68	0.39	0.00	1	0.9645
Compactness	0.72	0.09	0.70	0.08	1.06	1	0.3032
Convexity	0.93	0.06	0.96	0.05	2.20	1	0.1383
Form factor	0.66	0.13	0.65	0.10	0.12	1	0.7239
Roundness	0.53	0.12	0.50	0.11	0.00	1	1.0000
Solidity	0.91	0.07	0.88	0.08	2.97	1	0.0848
**Microglial processes**							
Process quantity	7.95	2.52	4.93	1.14	17.00	1	3.73×10^{-5}
Process nodes	44.33	33.64	10.67	5.73	8.76	1	0.0031
Process ends	55.53	37.15	15.93	6.43	9.73	1	0.0018
Total process length (µm)	613.80	288.52	197.42	77.63	12.11	1	5.01×10^{-4}
Mean process length (µm)	79.51	34.35	40.66	15.04	7.53	1	0.0061
Total process surface area (µm²)	971.90	636.46	423.80	167.15	5.15	1	0.0233
Mean process surface area (µm²)	125.36	81.93	89.54	38.21	1.21	1	0.2715
Total process volume (µm³)	138.80	110.42	82.47	40.74	1.78	1	0.1818
Mean process volume (µm³)	17.91	14.66	17.98	10.41	0.04	1	0.8466

Note. Likelihood-ratio statistics compare the random-intercept mixed model containing age with the corresponding model without the age term (df = 1). Numerical p values < 0.001 are shown in scientific notation.

Supplemental Table 4. Direct Fisher r-to-z comparisons of age-specific architecture–behavior coupling.

Values are animal-level Pearson correlations between finalized composite Dominant Scores (DS) and whisker-dependent texture discrimination (WTD) performance. Direct differences between the independent young and aged correlations were tested using Fisher's r-to-z transformation; age labels were not reassigned.

Architecture score	Young adult		Aged		Direct Fisher r-to-z comparison	
	n	r	n	r	Z	p
**Neuronal composite DS**	6	0.904	6	−0.378	2.320	0.02033
**Microglial composite DS**	10	0.910	9	−0.093	2.916	0.003548

Note. Z is the standardized difference between Fisher-transformed young and aged Pearson correlations, calculated using the corresponding group sample sizes. Positive Z values indicate stronger DS–WTD coupling in young than aged animals. All tests were two-tailed.

Supplemental Table 5. Age-accounted associations between PCA-derived architecture scores and whisker-dependent texture discrimination performance.

Animal-level WTD performance was evaluated against animal-balanced PCA-derived Dominant Scores (DS) across four architecture domains. Age-adjusted and age-interaction models are reported in parallel.

Architecture domain	n	Age-adjusted model		Age-interaction model	
		DS coefficient, b (95% CI)	p	DS × Age, b (95% CI)	p
Neuronal conventional morphology	12	0.0746 (−0.0252 to 0.1744)	0.1253	−0.1891 (−0.3474 to −0.0307)	**0.02495**
Neuronal spatial architecture	12	0.0075 (−0.0502 to 0.0653)	0.7742	−0.2078 (−0.3911 to −0.0244)	**0.03097**
Microglial conventional morphology	19	0.1067 (0.0522 to 0.1612)	**7.53×10^{-4}**	−0.0989 (−0.2223 to 0.0245)	0.1082
Microglial spatial architecture	19	0.0430 (0.0187 to 0.0673)	**0.00177**	−0.0538 (−0.1495 to 0.0420)	0.2500

Note. Animal-balanced PCA scores used inverse cell-count weighting ($1/n_i$ per cell). Models were WTD ~ DS + Age and WTD ~ DS × Age (Young = 0, Aged = 1). PC orientation was applied after PCA fitting and component retention; WTD was not used in score construction. DS × Age terms test age differences in domain-specific regression slopes; Figure 6 Fisher tests compare age-specific correlations for composite DS.

Supplemental Table 6. Animal-level associations between original morphometric variables and whisker-dependent texture discrimination performance.

For each variable, reconstructed cells were first averaged within animal, and Pearson correlations were then computed between the resulting animal-level morphometric values and whisker-dependent texture discrimination (WTD) performance within the corresponding neuronal or microglial cohort. Pearson r, 95% confidence intervals, and Holm-adjusted p values are reported for four domain-specific screening families.

A. Neuronal conventional morphology (n = 12 animals)

Morphometric variable	n	Pearson r	95% CI	Holm-adjusted p
Soma perimeter (µm)	12	−0.622	−0.881 to −0.074	0.5266
Soma area (µm²)	12	−0.619	−0.880 to −0.070	0.5266
Soma Feret maximum (µm)	12	−0.585	−0.867 to −0.016	0.5511
Soma Feret minimum (µm)	12	−0.723	−0.917 to −0.256	0.1723
Soma aspect ratio	12	−0.154	−0.669 to 0.461	1.0000
Soma compactness	12	0.250	−0.378 to 0.720	1.0000
Soma convexity	12	0.115	−0.491 to 0.646	1.0000
Soma form factor	12	0.097	−0.505 to 0.635	1.0000
Soma roundness	12	0.248	−0.380 to 0.720	1.0000
Soma solidity	12	0.234	−0.393 to 0.712	1.0000
Basal dendritic quantity	12	0.585	0.016 to 0.867	0.5511
Basal dendritic nodes	12	0.413	−0.211 to 0.798	1.0000
Basal dendritic ends	12	0.544	−0.044 to 0.852	0.6143
Basal total dendritic length (µm)	12	0.611	0.058 to 0.877	0.5266
Basal mean dendritic length (µm)	12	0.457	−0.159 to 0.817	1.0000
Basal total dendritic surface area (µm²)	12	0.775	0.362 to 0.934	0.0710
Basal mean dendritic surface area (µm²)	12	0.633	0.094 to 0.885	0.5098
Basal total dendritic volume (µm³)	12	0.687	0.187 to 0.904	0.2836
Basal mean dendritic volume (µm³)	12	0.554	−0.029 to 0.856	0.6143
Apical dendritic nodes	12	0.619	0.069 to 0.880	0.5266
Apical dendritic ends	12	0.595	0.032 to 0.871	0.5361
Apical total dendritic length (µm)	12	0.634	0.094 to 0.886	0.5098
Apical total dendritic surface area (µm²)	12	0.643	0.109 to 0.889	0.4845
Apical total dendritic volume (µm³)	12	0.794	0.405 to 0.940	**0.0488**

B. Neuronal spatial architecture (n = 12 animals)

Morphometric variable	n	Pearson r	95% CI	Holm-adjusted p
Convex hull area (µm²)	12	0.438	−0.182 to 0.809	0.6934
Convex hull perimeter (µm)	12	0.525	−0.071 to 0.844	0.5600
Convex hull volume (µm³)	12	0.705	0.220 to 0.910	0.1360
Convex hull surface area (µm²)	12	0.553	−0.031 to 0.855	0.5577
Basal proximal dendritic length (µm)	12	0.608	0.052 to 0.876	0.3959
Basal intermediate dendritic length (µm)	12	0.553	−0.030 to 0.856	0.5577
Basal distal dendritic length (µm)	12	0.328	−0.303 to 0.759	0.6934
Apical proximal dendritic length (µm)	12	0.627	0.083 to 0.883	0.3502
Apical intermediate dendritic length (µm)	12	0.426	−0.196 to 0.803	0.6934
Apical distal dendritic length (µm)	12	0.501	−0.102 to 0.835	0.5809
Apical orientation index	12	0.164	−0.453 to 0.674	0.6934
Basal orientation index	12	0.454	−0.163 to 0.815	0.6934
Oppositional polarity index	12	0.589	0.022 to 0.869	0.4403

C. Microglial conventional morphology (n = 19 animals)

Morphometric variable	n	Pearson r	95% CI	Holm-adjusted p
Soma perimeter (µm)	19	−0.542	−0.800 to −0.117	0.1643
Soma area (µm²)	19	−0.573	−0.815 to −0.160	0.1249
Soma Feret maximum (µm)	19	−0.578	−0.818 to −0.168	0.1237
Soma Feret minimum (µm)	19	−0.571	−0.814 to −0.158	0.1249
Soma aspect ratio	19	−0.008	−0.460 to 0.448	1.0000
Soma compactness	19	0.308	−0.170 to 0.668	1.0000
Soma convexity	19	−0.159	−0.572 to 0.318	1.0000
Soma form factor	19	0.222	−0.258 to 0.614	1.0000
Soma roundness	19	0.319	−0.158 to 0.675	1.0000
Soma solidity	19	0.493	0.051 to 0.774	0.2545
Process quantity	19	0.839	0.622 to 0.937	**1.33×10^{-4}**
Process nodes	19	0.690	0.343 to 0.871	**0.0173**
Process ends	19	0.718	0.391 to 0.884	**0.0092**
Total process length (µm)	19	0.822	0.587 to 0.929	**2.84×10^{-4}**
Mean process length (µm)	19	0.678	0.324 to 0.866	**0.0199**
Total process surface area (µm²)	19	0.686	0.337 to 0.869	**0.0176**
Mean process surface area (µm²)	19	0.434	−0.026 to 0.742	0.4454
Total process volume (µm³)	19	0.508	0.070 to 0.782	0.2379
Mean process volume (µm³)	19	0.137	−0.339 to 0.556	1.0000

D. Microglial spatial architecture (n = 19 animals)

Morphometric variable	n	Pearson r	95% CI	Holm-adjusted p
Convex hull area (µm²)	19	0.671	0.312 to 0.863	**0.0214**
Convex hull perimeter (µm)	19	0.605	0.208 to 0.831	0.0663
Convex hull volume (µm³)	19	0.769	0.484 to 0.907	**0.0020**
Convex hull surface area (µm²)	19	0.693	0.348 to 0.872	**0.0142**
Sholl intersections, 5 µm radius	19	0.587	0.181 to 0.822	0.0822
Sholl intersections, 10 µm radius	19	0.910	0.777 to 0.965	**1.81×10^{-6}**
Sholl intersections, 15 µm radius	19	0.867	0.681 to 0.948	**4.09×10^{-5}**
Sholl intersections, 20 µm radius	19	0.774	0.494 to 0.909	**0.0019**
Sholl intersections, 25 µm radius	19	0.782	0.508 to 0.912	**0.0015**
Sholl intersections, 30 µm radius	19	0.802	0.546 to 0.921	**8.11×10^{-4}**
Sholl intersections, 35 µm radius	19	0.766	0.479 to 0.905	**0.0021**
Sholl intersections, 40 µm radius	19	0.521	0.087 to 0.788	0.2002
Sholl intersections, 45 µm radius	19	0.336	−0.140 to 0.686	0.3884
Sholl intersections, 50 µm radius	19	0.414	−0.049 to 0.731	0.3884
Sholl intersections, 55 µm radius	19	0.397	−0.069 to 0.721	0.3884
Sholl intersections, 60 µm radius	19	0.259	−0.221 to 0.638	0.3884
Sholl process length, 5 µm radius (µm)	19	0.657	0.289 to 0.856	**0.0270**
Sholl process length, 10 µm radius (µm)	19	0.884	0.719 to 0.955	**1.37×10^{-5}**
Sholl process length, 15 µm radius (µm)	19	0.856	0.658 to 0.944	**7.21×10^{-5}**
Sholl process length, 20 µm radius (µm)	19	0.834	0.612 to 0.934	**2.18×10^{-4}**
Sholl process length, 25 µm radius (µm)	19	0.802	0.546 to 0.921	**8.11×10^{-4}**
Sholl process length, 30 µm radius (µm)	19	0.815	0.573 to 0.926	**4.93×10^{-4}**
Sholl process length, 35 µm radius (µm)	19	0.771	0.487 to 0.907	**0.0020**
Sholl process length, 40 µm radius (µm)	19	0.701	0.363 to 0.876	**0.0123**
Sholl process length, 45 µm radius (µm)	19	0.500	0.059 to 0.777	0.2348
Sholl process length, 50 µm radius (µm)	19	0.402	−0.064 to 0.724	0.3884
Sholl process length, 55 µm radius (µm)	19	0.452	−0.003 to 0.752	0.3122
Sholl process length, 60 µm radius (µm)	19	0.468	0.018 to 0.761	0.3028

Note. Holm adjustment was applied separately within each of the four domain-specific families. Bold values in the Holm-adjusted p column indicate $p < 0.05$ after correction. Numerical p values < 0.001 are shown in scientific notation. The constant apical dendritic quantity variable was excluded from the neuronal conventional family, consistent with the PCA input dataset.
